# A positive feedback circuit driven by m^6^A-modified circular RNA facilitates colorectal cancer liver metastasis

**DOI:** 10.1186/s12943-023-01848-1

**Published:** 2023-12-13

**Authors:** Kaixuan Zeng, Jianhong Peng, Yue Xing, Linjie Zhang, Peishan Zeng, Weihao Li, Weili Zhang, Zhizhong Pan, Chi Zhou, Junzhong Lin

**Affiliations:** 1 Precision Medical Research Institute, the Second Affiliated Hospital of Xi’an Jiaotong University, Xi’an, 710000 China; 2 Department of Colorectal Surgery, Sun Yat-sen University Cancer Center, Guangzhou, 510060 China; 3 State Key Laboratory of Oncology in South China, Collaborative Innovation Center for Cancer Medicine, Sun Yat-sen University Cancer Center, Guangzhou, 510060 Guangzhou China; 4 Guangdong Provincial Key Laboratory of Malignant Tumor Epigenetics and Gene Regulation, Medical Research Center, Sun Yat-Sen Memorial Hospital, Sun Yat-Sen University, Guangzhou, 510120 China; 5 Breast Tumor Center, Sun Yat-Sen Memorial Hospital, Sun Yat-Sen University, Guangzhou, 510120 China; 6 Department of Rehabilitation Medicine, The Third Affiliated Hospital, Sun Yat-Sen University, Guangzhou, 510630 China

**Keywords:** Circular RNA, Translation, N^6^-methyladenosine, Hippo signaling, Biomarker

## Abstract

**Background:**

Liver metastasis is the leading cause of death in patients with colorectal cancer (CRC). Emerge evidence suggests that circular RNA (circRNA) is a pivotal player in cancer progression. However, its role in CRC liver metastasis remains largely unknown.

**Methods:**

Circ-YAP expression was detected by qRT-PCR and in situ hybridization. The function of circ-YAP was tested by wound healing, transwell and CCK-8 assays. RNA immunoprecipitation, pull-down, luciferase reporter, chromatin immunoprecipitation assays were used to investigate the mechanism underlying circ-YAP promoting CRC liver metastasis. CRC liver metastasis animal model was established to assess the effect of circ-YAP in vivo.

**Results:**

Circ-YAP was notably upregulated in CRC with liver metastasis, which was associated with dismal prognosis. Circ-YAP promoted CRC cell migration and invasion in vitro, and facilitated liver metastasis in patient-derived xenografts (PDX) models in vivo. Mechanistically, circ-YAP encoded a novel truncated protein containing 220 amino acids, termed as YAP-220aa, which competitively bound to LATS1, resulting in YAP dephosphorylation and nuclear translocation, thereby activating a cohort of metastasis-promoting genes. Importantly, N^6^-methyladenosine (m^6^A) modification orchestrated efficient initiation of circ-YAP translation, requiring m^6^A reader YTHDF3 and eIF4G2 translation initiation complex. Intriguingly, circ-YAP was transcriptionally enhanced by YAP/TEAD complex, thus forming a positive regulatory feed-forward loop.

**Conclusions:**

Our findings reveal a previously uncharacterized oncoprotein encoded by circ-YAP, implying a promising biomarker and therapeutic target for CRC patients with liver metastasis.

**Supplementary Information:**

The online version contains supplementary material available at 10.1186/s12943-023-01848-1.

## Background

Colorectal cancer (CRC) is one of the most common malignant tumors of the digestive tract, with the incidence and mortality ranking the third and second among all malignant tumors, respectively [1]. Tumor metastasis is the main cause of death of CRC patients. Clinically, about 45–60% of CRC patients have liver metastasis, and more than 90% of liver metastases cannot be initially resected [2]. The median survival time of CRC patients with liver metastases without surgical treatment is only 6.9 months, and the 5-year survival rate is less than 5% [3]. Therefore, it is of great clinical significance to decipher the mechanism underlying CRC liver metastasis and discover new therapeutic targets for the prevention and intervention of metastasis to improve the survival rate of patients.

Circular RNA (circRNA) is a special class of endogenous RNA molecules, with a covalently closed loop structure, without the traditional 5’-end “cap structure” and 3’-end poly A tail [4]. It is generated by a spliceosome-catalyzed back-splicing event, and one genetic locus can produce one or more circRNAs [5]. High-throughput sequencing and *in silico* approaches have identified that circRNA is highly conserved and widely expressed in a disease-, tissue- or cell-specific pattern [6]. Increasingly, circRNA is being implicated in the development and progression of various human diseases, and some circRNAs are identified as available biomarkers for predicting disease progression and prognosis [7, 8]. The potential mechanism by which circRNA functions is complicated [9], the most of which is proposed to act as “miRNA sponge”, with CDR1as having more than 70 miR-7 binding sites as the typical representative [10, 11]. Moreover, several circRNAs have been identified to directly bind to functional proteins, acting as scaffolds or decoys involved in gene regulation [12]. Interestingly, some recent evidence indicates that circRNA is able to be translated into the functional peptides [13, 14]. For instance, Nlgn-173aa encoded by circ-Nlgn was proposed as a novel transcription factor that promoted myocardial fibrosis [15]. In addition, the circRNA-encoded truncated proteins such as E-Cad-254aa and ARHGAP35-1289aa have been reported as pivotal players in glioma [16]and hepatocellular carcinoma [17], respectively. Due to the head-to-tail shape, circRNA encodes protein in a cap-independent manner, which is driven by internal ribosome entry site (IRES) or N6-methyladenosine (m^6^A) modification [18]. m^6^A is the most abundant internal modification of eukaryotic RNA, which is dynamically reversible and catalyzed by m^6^A writer (METTL3/14 and WTAP), removed by m^6^A eraser (FTO and ALKBH5) and recognized by m^6^A reader (YTHDF1/2/3 and YTHDC1/2) [19]. To date, only a few of these small endogenous proteins hidden in circRNAs have been characterized, the functional relevance of the vast majority is yet to be found.

In this study, we identified a circRNA, circ-YAP, as a driver of CRC liver metastasis. Circ-YAP contains a 220-aa open reading frame (ORF) and encodes a novel YAP protein isoform, termed as YAP-220aa, in an m^6^A-dependent manner. Further, we found that YAP-220aa activated YAP signaling via preventing LATS1-mediated YAP phosphorylation and cytoplasmic retention.

## Materials and methods

### CRC tissues and cell lines

All CRC tissues included in this study were collected from Sun Yat-sen University Cancer Center, including fresh frozen (30 normal cases, 23 CRC cases, 17 CRC with liver metastasis, 9 cases with lung metastasis and 7 cases with brain metastasis) and paraffin embedded (25 normal cases, 211 CRC cases and 56 CRC with liver metastasis) tissues. Written informed consent was obtained from each subject. This study was approved by the Institutional Ethical Review Boards of Sun Yat-sen University Cancer Center (SL-B2022-276-02). The normal FHC cells (CRL-1831) and CRC cells including HT-29 (HTB-38), SW480 (CCL-228), DLD1 (CCL-221), HCT116 (CCL-247), SW620 (CCL-227) and LoVo (CCL-229) were obtained from ATCC. The above cells were cultured in DMEM medium with 10% fetal bovine serum (FBS). Besides, TC71 PDX cells were obtained from XENTECH (No. XTM-233_CXT-399/R5700), and cultured in advanced DMEM/F12 medium supplemented with 8% FBS, 1% antibiotics and 1% glutamin. All cells were periodically tested for mycoplasma contamination.

### Quantitative real-time polymerase chain reaction (qRT-PCR)

Total RNA was isolated using Trizol reagent (Invitrogen, CA, USA), then, 1 μg RNA was reverse transcribed into cDNA using PrimeScript RT Enzyme (Takara Bio, Dalian, China). RNA amplification and quantification were carried out with TB Green Premix Ex Taq II Kit (Takara Bio). The specificity of all primers was verified by the melt curve and the sequences are listed in Table S1.

### Fluorescence in situ hybridization (FISH), ISH and immunohistochemisty (IHC)

The FAM-labeled probe targeting the junction site of circ-YAP was designed and synthesized by GenePharma (Shanghai, China), followed by hybridization using the FISH Kit according to the manufacturer’s instructions (GenePharma). For ISH assay, the paraffin-embedded tissues were digested with proteinase K, and incubated with 5’-digoxin-labeled probe against circ-YAP junction site at 55 °C overnight. After incubation with anti-digoxin antibody (Roche, Basel, Switzerland) at 4 °C overnight, the slides were stained with NBT/BCIP reagent. For IHC staining, the anti-YAP-220aa (produced by GenScript) and anti-YAP (#14,074, CST) antibodies were used with the dilution ratio of 1:50 and 1:400, respectively. The protein signals were visualized by DAB solution. The semi-quantitative analysis of ISH/IHC staining was conducted using H-score method as previously described [20].

### Vectors, oligonucleotides and transfection

To silence circ-YAP, the CRISPR/Cas13d system was used [21]. In brief, three sgRNAs targeting the junction site of circ-YAP were designed and synthesized (gRNA#1: TCCTTTCCTTAACAGGCCAGTACTGATGCA; gRNA#2: TCAGATCCTTTCCTTAACAGGCCAGTACTG; gRNA#3: TCCTTAACAGGCCAGTACTGATGCAGGCAC), followed by insertion into pLKO.1 vector containing direct repeats of RfxCas13d. Lentivirus production was conducted using psPAX2 and pMD2.G vector, followed by infection into CRC cells with 5 mg/mL polybrene and screening with 1.5 μg/mL puromycin. To construct circ-YAP expression vector, the full-length of circ-YAP was synthesized and inserted into pLV-circ-Puro vector containing reverse complementary sequences on both sides. To knockout of YTHDF3, three sgRNAs targeting YTHDF3 (gRNA#1: CTAAGCGAATATGCCGTAAT; gRNA#2: GTGGACTATAATGCGTATGC; gRNA#3: AAAGTTGACTCTTCTCGTAA) were inserted into CRISPR/Cas9 All-in-One lentiviral vector, followed by infection into cells and selection of single clone. For constructing shRNA expression vector (sh-YAP targeting YAP 3`-UTR: CCCAGTTAAATGTTCACCAAT), the pLKO.1 lentiviral vector was used, followed by infection and puromycin selection. Besides, siRNAs targeting eIF4G2 and TEADs were commercially purchased from Ribobio (Guangzhou, China). YAP-5SA and YAP-5SA/S94A were obtained from Addgene. Mutation of m^6^A motif was conducted using Q5 Site-directed Mutagenesis Kit (New England Biolabs, CA, USA) according to the manufacturer’s instructions. All constructs were confirmed by Sanger sequencing. Cell transfection was carried out using Lipofectamine 3000 (Invitrogen) and the transfection efficiency was tested by qRT-PCR or western blot assays.

### Wound healing, transwell and CCK-8 assays

Cell migration was tested by wound healing assay. Briefly, CRC cells were plated into 6-well plates, and the scratches were generated using a sterile pipette tip. Then, cells were cultured in DMEM medium without FBS. After 24 h, the migration distance was recorded. For detection of cell invasion, the upper chamber with an 8 mm pore size filter (BD Falcon, CA, USA) was used and the bottom chamber was filled with 600μL DMEM complete medium. After 24 h, the invaded cells were stained by crystal violet. For CCK-8 assay, CRC cells were plated into 96-well plates, followed by incubation with 10μL CCK-8 reagent (Dojindo, Kumamoto, Japan) at 37 °C for 2 h. The absorbance at 450 nm of each well was recorded with a microplate reader.

### Establishment of CRC liver metastasis model

For the spontaneous liver metastasis model, TC71 PDX cells with or without circ-YAP knockdown were harvested by trypsinization and washed three times with cold PBS, followed by orthotopically injection of 50μL 3 × 10^6^ cell suspension into the colonic subserosa of NOD/SCID mice. After 10 weeks, mice were euthanized, and liver tissues were carefully dissected out to detect for metastatic lesions. For the experimental liver metastasis model, 20μL 1 × 10^6^ TC71 PDX cells suspended in PBS were slowly injected into spleen, after 4 weeks, the number of liver metastatic nodule in each group was counted. Liver metastasis burden was defined as the number of metastatic nodules multiplied by the diameter of the metastatic lesions. All procedures for animal experiments were approved by the Institutional Animal Care and Use Committee of Sun Yat-sen University (SYSU-IACUC-2021-000653).

### Western blot and co-immunoprecipitation (Co-IP)

Cells were washed by cold PBS and lysed by RIPA buffer supplemented with 1×protease inhibitor cocktail (Roche). The concentration of protein was detected by Pierce™ BCA Protein Quantification Kit (Invitrogen). Equal amount of protein was loaded onto 8-10% SDS-PAGE gel and transferred onto PVDF membrane. Then, the membrane was incubated with appropriate primary and secondary antibodies, and visualized using Pierce™ ECL Western solution (Invitrogen). For Co-IP assay, cell lysates were pre-cleared by incubating with 20μL protein A/G agarose (Gibco, CA, USA). After that, the supernatant was incubated with appropriate primary antibody at 4 °C for 3 h, followed by incubation with 40μL protein A/G agarose at 4 °C for 30 min. The enriched proteins were separated by SDS-PAGE gel and analyzed by western blot. The primary antibodies used in this study were as follows: anti-Flag (#80010-1-RR, Proteintech), anti-YAP-220aa (provided by Genescript), anti-METTL3 (#15073-1-AP, Proteintech), anti-YTHDF1 (#ab220162, Abcam), anti-YTHDF2 (#ab220163, Abcam), anti-YTHDF3 (#25537-1-AP, Proteintech), anti-eIF4G2 (#5169, CST), anti-eIF4A (#2013, CST), anti-eIF4B (#13,088, CST), anti-LATS1 (#3477, CST), anti-YAP (#14,074, CST), anti-14-3-3 (#9640, CST), p-YAP (S127) (#13,008, CST), anti-TEAD1 (#12,292, CST), anti-Tubulin (#11224-1-AP, Proteintech), anti-CDX2 (##ab76541, Abcam), anti-GAPDH (#60004-1-Ig, Proteintech) and anti-Histone H3 (#ab1791, Abcam).

### Immunofluorescence (IF)

CRC cells were fixed with 4% paraformaldehyde, permeabilized with 0.1% Triton X-100 and blocked using 5% BSA solution for 30 min at room temperature.Then, cells were incubated with anti-Flag, anti-YAP-220aa and anti-YAP antibodies overnight at 4 °C. After incubating with fluorescein-conjugated secondary antibody for 1 h, the fluorescence signal was observed using a fluorescence microscope. Cell nucleus was stained with DAPI solution.

### RNA immunoprecipitation (RIP) and methylated immunoprecipitation (meRIP)

RIP assay was conducted using the Magna RIP Kit (#17–700, Millipore, MA, USA) as per the manufacturer’s protocols. In short, the magnetic beads pre-coated with anti-YTHDF3 or anti-IgG (Millipore) were incubated with CRC cell lysates at 4 °C for 12 h. After protein digestion by proteinase K, the enriched RNA was extracted by Trizol reagent, followed by qRT-PCR analysis of circ-YAP level. For meRIP assay, the Magna MeRIP™ m^6^A Kit (17 − 10,499, Millipore) was used. Total RNA was fragmented into 100nt or less for 5 min at 70 °C, followed by incubation with anti-m^6^A antibody (#MABE1006, Millipore) and Magna Protein A/G Magnetic Beads overnight at 4 °C. The enriched RNA was eluted by m^6^A 5′-monophosphate sodium salt, followed by RNA extraction and qRT-PCR analysis.

### RNA pull-down assay

For in vivo pull-down assay, the biotin-labeled probe targeting circ-YAP junction site was synthesized and incubated with CRC cell lysates at 4 °C for 5 h, followed by incubation with the Streptavidin Magnetic Beads (Invitrogen) at 4 °C for 1 h. Then, the enriched proteins were eluted for western blot analysis. For in vitro pull-down assay, the linear circ-YAP was in vitro transcribed using the T7 Transcription Kit (Invitrogen) and labeled with biotin using Biotin RNA Labeling Mix (Roche), followed by circularization using T4 RNA ligase I. The above synthesized circ-YAP was incubated with recombinant human YTHDF3 protein (#ab166020, Abcam) in binding buffer (20mM Tris, 150mM NaCl, 1% Triton X-100, 2mM DTT, 1mM EDTA) at 4 °C for 1 h, followed by incubation with the Streptavidin Magnetic Beads (Invitrogen) and western blot analysis.

### Detection of nascent circ-YAP

Cells were incubated with 5,6-dichlor-obenzimidazole 1-β-D-ribofuranoside (DRB; Sigma-Aldrich, MO, USA) to block transcription. After DRB release, the newly transcribed RNA was labeled with 4-thiouridine (4sU; Sigma). Total RNA was extracted using Trizol reagent, followed by biotinylation and pull-down with the Streptavidin Magnetic Beads (Invitrogen). The nascent RNA was serially washed and subjected for qRT-PCR analysis of circ-YAP level.

### Luciferase reporter assay

For detecting YAP transcription activity, cells with circ-YAP silencing or overexpression were transfected with YAP luciferase reporter (8xGTIIC-luciferase, #34,615, Promega) using Lipofectamine 3000 (Invitrogen). After 48 h of transfection, the relative luciferase activity was detected by the dual-luciferase reporter system (Promega) according to the manufacturer’s instructions. For analysis of circ-YAP promoter activity, the full-length or truncated circ-YAP promoter was inserted into pGL3-basic vector (Promega), followed by co-transfection with pRL-TK and YAP-5SA vectors into cells using Lipofectamine 3000 (Invitrogen). The relative luciferase activity was tested as mentioned above. Each group was run in triplicate in 48-well plates.

### Chromatin immunoprecipitation (ChIP) and re-ChIP

ChIP assay was carried out using the commercialized SimpleChIP® Plus Sonication Chromatin IP Kit (#56,383, CST) based on the manufacturer’s protocols. The used ChIP-grade antibodies are as following: anti-YAP (#14,074, CST), anti-PoI II (#N-20, Santa Cruz biotechnology) and anti-p-PoI II (S5) (#ab5408, Abcam). For re-ChIP assay, the DNA complexes were immunoprecipitated and eluted in the first-step ChIP using anti-YAP (#14,074, CST) antibody, followed by addition into 10mM DTT and incubation for 30 min at 37 °C. After centrifugation, the supernatant was diluted and immunoprecipitated using anti-TEAD1 antibody (#610,922, BD Biosciences). The resulting precipitated DNA samples were subjected for qPCR analysis.

### DNA pull-down assay

The biotinylated probe targeting circ-YAP promoter was designed and synthesized, followed by incubation with the sonicated nuclear extracts at 4 °C overnight with agitation. The Streptavidin Magnetic Beads (Invitrogen) was added and incubated for 1 h at 4 °C. The beads were washed three times and the enriched proteins were eluted by 1×loading buffer, followed by western blot analysis of YAP and TEAD1 levels.

### Statistical analysis

Data are shown as mean ± standard deviation (SD). Two-tailed Student’s t-test was used to compare the results for any two preselected groups accounting for variance. The ROC curve was used to estimate the predictive accuracy. The survival curve of CRC patients was generated using Kaplan-Meier plotter, which was analyzed by Log-rank test. All statistical charts were generated by Graph Prism 7 software (La Jolla, CA, USA). A probability value less than 0.05 was considered statistically significant.

## Results

### Circ-YAP is linked to CRC liver metastasis

**Fig. 1:**
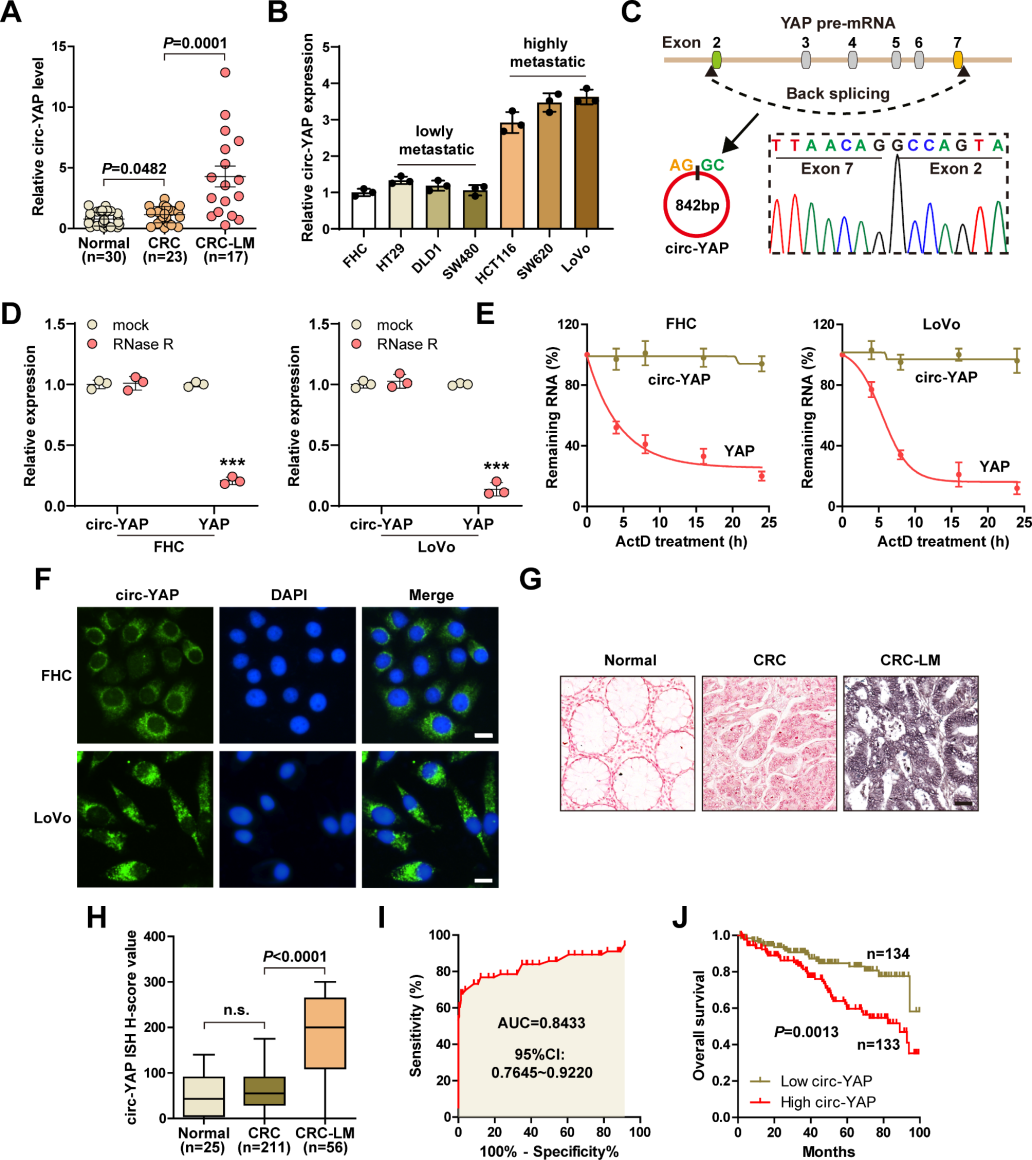
Identification of circ-YAP in CRC liver metastasis tissues. **A**, **B**. qRT-PCR analysis of circ-YAP expression in CRC tissues and cell lines. **C**. Sanger sequencing verifying the junction site of circ-YAP. **D**, **E**. Cells were treated with 3U/μg of RNase R or 5 μg/ml Actinomycin D, followed by qRT-PCR analysis of circ-YAP and YAP mRNA levels. **F**. FISH assay detecting the location of circ-YAP, DAPI was used to stain cell nucleus. Scale bar, 25 μm. **G**-**I**. ISH staining detecting circ-YAP expression in paraffin embedded tissues (**G**, **H**), followed by ROC curve analysis of the predictive accuracy (**I**). The dark purple denotes positive staining of circ-YAP. Scale bar, 50 μm. **J**. The survival curve of CRC patients with low and high circ-YAP levels. ***P < 0.001. Data (B, D, E) are the mean ± SD of three independent experiments carried out in triplicate

To identify the key circRNAs with translational activity in CRC liver metastasis, we analyzed circRNA microarray and ribosome nascent-chain complex-bound RNA sequencing data. The overlapped results showed that circ-YAP was upregulated in CRC liver metastasis and might have translation potential (Figure S1A, B). Moreover, circ-YAP was closely related to liver specific gene set, as demonstrated by GSEA enrichment analysis (Figure S1C). Next, we tested circ-YAP expression in fresh frozen tissues, the results showed that as compared to normal tissues, circ-YAP was slightly increased in CRC tissues, while markedly overexpressed in cases with liver metastasis (Fig. 1A). However, no significant differences were observed between circ-YAP expression and CRC lung or brain metastasis (Figure S1D). Likewise, high circ-YAP was observed in CRC cell lines with high metastatic potential (Fig. 1B). The sequence analysis revealed that circ-YAP was originated from the back-splicing of exon 2 and 7 of YAP pre-mRNA, the mature full-length was 842 bp (Fig. 1C). Circ-YAP, but not its linear isoform, was resistant to RNase R digestion in both normal and CRC cells (Fig. 1D, Figure S1E, F). Furthermore, the half-life of circ-YAP exceeded 24 h (Fig. 1E, Figure S1G). The results of qRT-PCR and FISH assays showed that circ-YAP was mainly located in the cytoplasm (Fig. 1F, Figure S1I, J). To further explore the clinical relevance of circ-YAP, we collected paraffin embedded tissues and detected circ-YAP expression using ISH assay. As expected, circ-YAP was significantly upregulated in CRC tissues with liver metastasis (Fig. 1G, H), with an area under curve (AUC) value of 0.8433 (95% CI: 0.7645–0.9220) (Fig. 1I). More importantly, patients with high circ-YAP had shorter survival time than those with low circ-YAP (Fig. 1J). In addition, the expression of circ-YAP was almost unaffected by acid-base conditions and repeated freeze-thawing (Figure S1K-M). In sum, these data suggest that circ-YAP is a bona fide circRNA that may be used as a promising indicator and prognostic marker of CRC liver metastasis.

### Knockdown of circ-YAP alleviates CRC liver metastasis burden

**Fig. 2:**
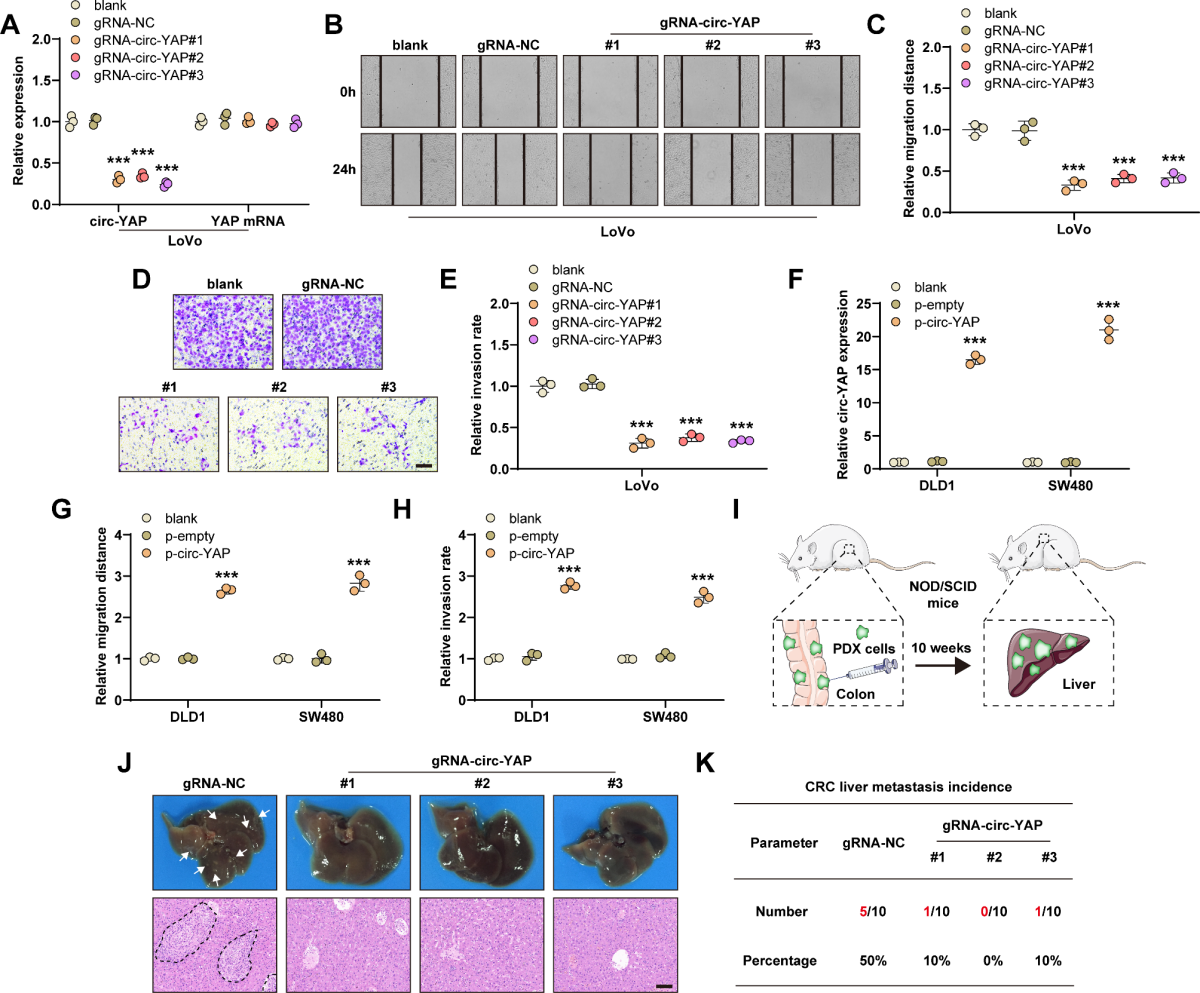
Silencing of circ-YAP alleviates CRC liver metastasis burden. **A**. qRT-PCR verifying the knockdown efficiency of the designed sgRNAs. **B**, **C**. Wound healing assay testing cell migration in circ-YAP-silenced LoVo cells. **D**, **E**. Transwell assay testing cell invasion in circ-YAP-silenced CRC cells. Scale bar, 100 μm. **F**. qRT-PCR verifying the overexpression efficiency of circ-YAP. **G**, **H**. Cell migration and invasion were tested in DLD1 and SW480 cells after circ-YAP overexpression. **I**. The sketch showing the establishment of the spontaneous liver metastasis model. **J**. The representative images of CRC liver metastasis in the indicated groups. Scale bar, 100 μm. **K**. The incidence of CRC liver metastasis in each group. ***P < 0.001. Data (A, C, E, F, G, H) are the mean ± SD of three independent experiments carried out in triplicate

To investigate the biological functions of circ-YAP, we used CRISPR/Cas13d technology to silence circ-YAP (Figure S2A). As shown in Fig. 2A, all three designed gRNAs could effectively knock down circ-YAP, but did not affect YAP mRNA expression. The wound healing and transwell assays showed that silencing of circ-YAP significantly inhibited LoVo cell migration (Fig. 2B, C) and invasion (Fig. 2D, E), respectively. And depletion of circ-YAP resulted in same effects in SW620 cells (Figure S2B). Next, we overexpressed circ-YAP in CRC cells with low circ-YAP expression (Fig. 2F), the results displayed that the ability of cell migration and invasion was significantly enhanced after circ-YAP overexpression (Fig. 2G, H). However, manipulation of circ-YAP did not affect cell viability, as shown by CCK-8 assay (Figure S2C). The spontaneous CRC liver metastasis PDX model was established via injection of TC71 PDX cells into the colon wall of NOD/SCID mice (Fig. 2I). Ten weeks later, 50% mice in control group, but only 6.7% mice in circ-YAP-silenced group developed liver metastases (Fig. 2J, K). In addition, we also established the experimental liver metastasis model via injection of TC71 cells into spleen (Figure S2D), the results showed that circ-YAP knockdown attenuated liver metastasis burden (Figure S2E). Further, we performed IHC staining of CDX2, an intestine-specific nuclear transcription factor, the results showed that the metastasized cells were indeed CRC cells but not hepatocytes or immune cells (Figure S2E). Collectively, these data indicate that circ-YAP plays a crucial role in CRC cell aggressiveness and liver metastasis.

### Circ-YAP encodes YAP protein isoform YAP-220aa

**Fig. 3:**
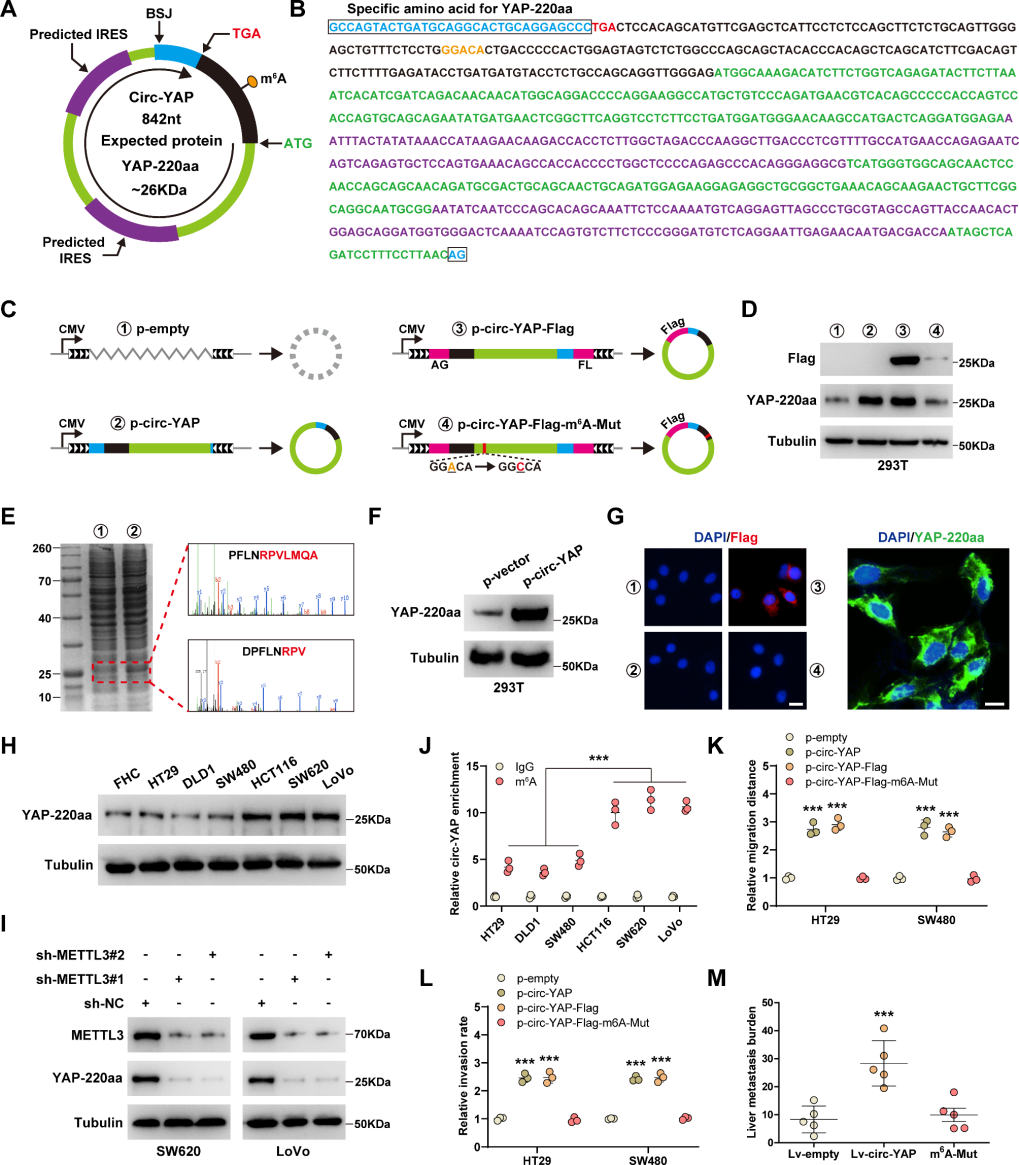
Circ-YAP encodes YAP-220aa mediated by m^6^A. **A**, **B**. The sketch and full-length sequence of circ-YAP. **C**. The sketch showing the construction of the indicated vectors. **D**. Western blot analysis of Flag and YAP-220aa protein levels in 293T cells transfected with the above vectors. **E**. Coomassie blue staining of protein samples from control and circ-YAP-overexpressing cells, followed by mass spectrometry of the indicated gels. **F**. Western blot analysis of YAP-220aa protein levels in circ-YAP-overexpressing cells. **G**. IF staining of Flag and YAP-220aa in 293T cells. Scale bar, 25 μm. **H**. Western blot analysis of YAP-220aa protein levels in CRC cell lines. **I**. Western blot testing the effect of METTL3 knockdown on YAP-220aa expression. **J**. meRIP assay testing the m^6^A levels on circ-YAP in CRC cells. **K**, **L**. Cell migration and invasion in HT29 and SW480 cells transfected with the indicated vectors. **M**. The experimental liver metastasis model testing the effect of circ-YAP or circ-YAP-m^6^A-mutation on CRC liver metastasis (n = 5 per group). ***P < 0.001. Data (J, K, L) are the mean ± SD of three independent experiments carried out in triplicate. The uncropped western blot data are provided as a Original Blot Image file

By sequence alignment, we found that circ-YAP contained a potential ORF spanning junction site that encoded a 220-aa protein (Fig. 3A, B). To verify that circ-YAP was translatable, we added Flag tag into circ-YAP-overexpressing vector; specifically, the junction site of circ-YAP was moved to the stop codon of the ORF, and the Flag sequence was divided into two by one, locating on both sides of circ-YAP (Fig. 3C). After transfection into HEK293T cells, an approximately 26 kDa band was detected by anti-Flag tag antibody (Fig. 3D). Moreover, this band was more obvious after circ-YAP overexpression, as shown by coomassie blue staining, subsequently, the unique amino acid sequence of YAP-220aa was verified by mass spectrometry analysis (Fig. 3E). Next, we generated a rabbit polyclonal antibody specifically targeting YAP-220aa, and confirmed that it could effectively detect the endogenous YAP-220aa protein (Fig. 3F). Given that circRNA translation is driven by IRES or m^6^A modification, we first evaluated the translational activity of IRES on circ-YAP. Two putative IRES sequences were inserted into dual luciferase vector system (Figure S3A), the result showed that the Luc/Rluc activity was unaltered in IRES vector as compared to empty vector (Figure S3B), indicating that IRES-driven translation is not responsible for YAP-220aa generation. Of note, a highly conserved m^6^A site “GGACA” was found proximal to the translation initiation site (Fig. 3B). After mutation of adenine into cytosine (Fig. 3C), YAP-220aa protein was almost undetected by anti-Flag antibody (Fig. 3D), implying that m^6^A is essential for circ-YAP translation. YAP-220aa was dominantly localized in the cytoplasm (Fig. 3G, Figure S3C), and was overexpressed in highly metastatic CRC cells (Fig. 3H). Consistently, silencing of METTL3, an m^6^A writer, significantly reduced endogenous YAP-220aa expression (Fig. 3I), and more m^6^A enrichment on circ-YAP was observed in highly metastatic CRC cells compared to lowly metastatic cells (Fig. 3J). Functionally, overexpression of circ-YAP enhanced the migration and invasion of HT29 and SW480 cells, where these effects were blocked by m^6^A mutation (Fig. 3K, L). Moreover, the experimental liver metastasis model showed that wild-type circ-YAP, but not m^6^A-mutated one, increased liver metastasis of CRC cells in vivo (Fig. 3M). Altogether, these findings demonstrate that circ-YAP functions through translating into a novel YAP protein isoform, which is mediated by m^6^A modification.

### m^6^A reader YTHDF3 is required for circ-YAP translation

**Fig. 4:**
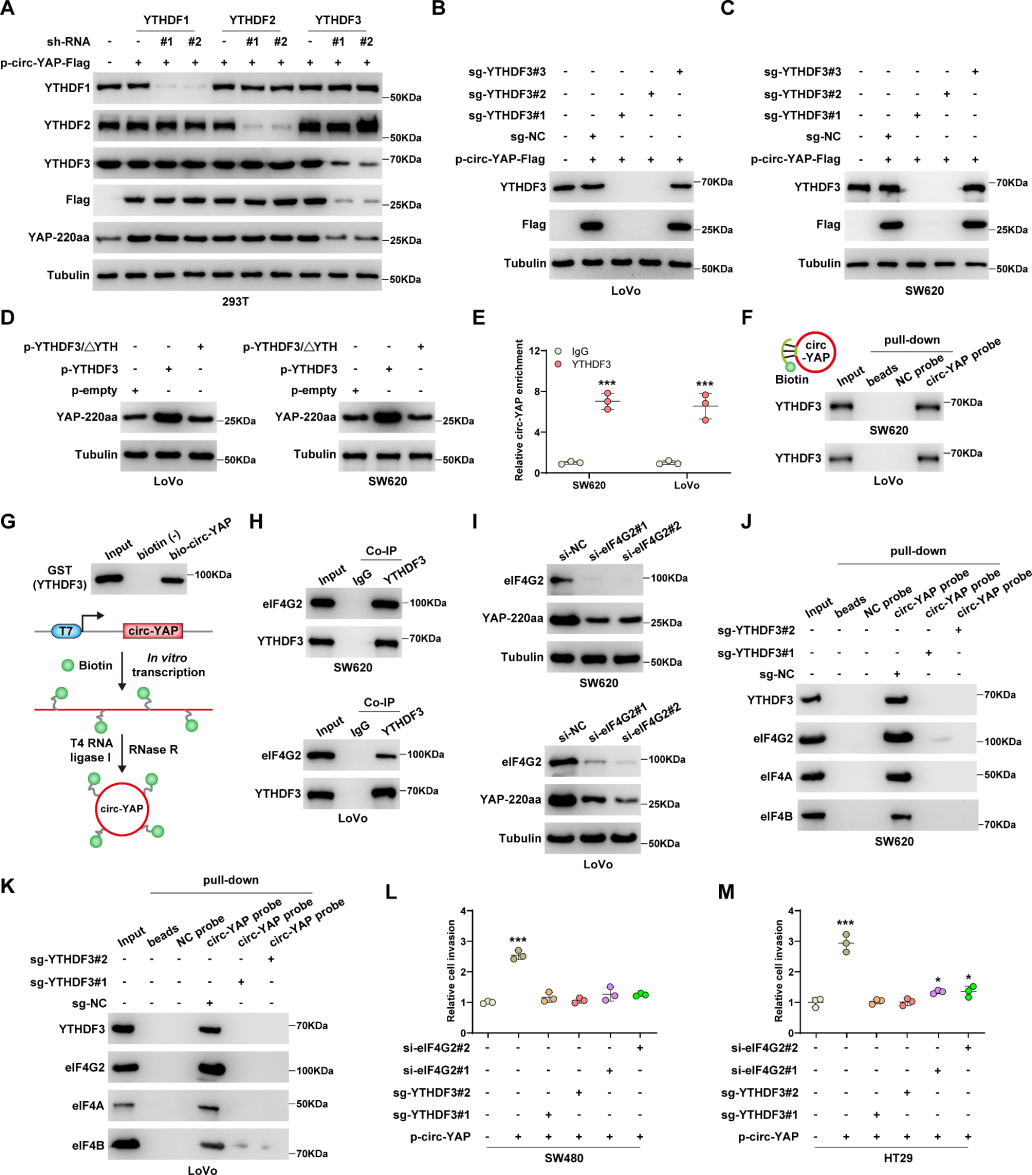
YTHDF3 recruits eIF4G2 complex to driver circ-YAP translation. **A**. Western blot testing the levels of the indicated proteins in circ-YAP-overexpressing 293T cells after knockdown of YTHDF1/2/3. **B**, **C**. Western blot testing the effect of YTHDF3 knockout on YAP-220 level. **D**. The indicated vectors were transfected into LoVo and SW620 cells, followed by western blot analysis of YAP-220 level. **E**. RIP assay using anti-YTHDF3 antibody, followed by qRT-PCR analysis of circ-YAP enrichment. **F**, **G**. The in vivo and in vitro pull-down assays using biotin-labeled circ-YAP probes, followed by western blot analysis of YTHDF3 level. **H**. Co-IP assay testing the interaction between YTHDF3 and eIF4G2 proteins. **I**. Western blot testing the effect of eIF4G2 silencing on YAP-220aa expression. **J**, **K**. RNA pull-down assays using biotin-labeled circ-YAP probes in YTHDF3^−/−^ SW620 and LoVo cells, followed by western blot analysis of the indicated protein levels. **L**, **M**. Transwell assay testing cell invasion in circ-YAP-overexpressing SW480 and HT29 cells after YTHDF3 knockout or eIF4G2 silencing. *P < 0.05, ***P < 0.001. Data (E, L, M) are the mean ± SD of three independent experiments carried out in triplicate. The uncropped western blot data are provided as a Original Blot Image file

We wondered which m^6^A reader proteins participated in the process of circ-YAP translation. As shown in Fig. 4A, knockdown of YTHDF3, not YTHDF1/2, significantly blocked the increased YAP-220aa levels caused by circ-YAP overexpression (Fig. 4A). However, the expression of circ-YAP was not affected by YTHDF1/2/3 (Figure S4A-C). To further verify the effect of YTHDF3 on circ-YAP translation, we constructed YTHDF3 knockout CRC cell lines using CRISPR/Cas9 technology. The results showed that the translation product of p-circ-YAP-Flag was undetectable in YTHDF3^−/−^ LoVo and SW620 cells (Fig. 4B, C). Conversely, YAP-220aa expression was notably increased in YTHDF3-overexpressing cells, but this effect was disappeared after deletion of the active YTH domain (Fig. 4D). The results of RIP and RNA pull-down assays revealed the interaction between circ-YAP and YTHDF3 (Fig. 4E, F). Moreover, circ-YAP was in vitro synthesized and incubated with GST-tag YTHDF3 protein, the results displayed that circ-YAP directly binds to YTHDF3 (Fig. 4G). Given that the non-canonical eIF4G2 is required for YTHDF3-mediated translational regulation [22], we conducted Co-IP assay and confirmed that YTHDF3 and eIF4G2 form an endogenous complex in both SW620 and LoVo cells (Fig. 4H). As expected, silencing of eIF4G2 dramatically decreased YAP-220aa protein levels (Fig. 4I). The results of RNA pull-down assay showed that eIF4G2 and eIF4A/B were enriched by circ-YAP, whereas these interactions were abolished by YTHDF3 knockout (Fig. 4J, K), indicating that circ-YAP recruits eIF4G2 translation initiation complex via YTHDF3. Functionally, the enhanced cell invasion caused by circ-YAP was evidently counteracted after knockout of YTHDF3 or silencing of eIF4G2 (Fig. 4L, M).

### YAP-220aa interacts with LATS1 and increases YAP nuclear translocation

**Fig. 5:**
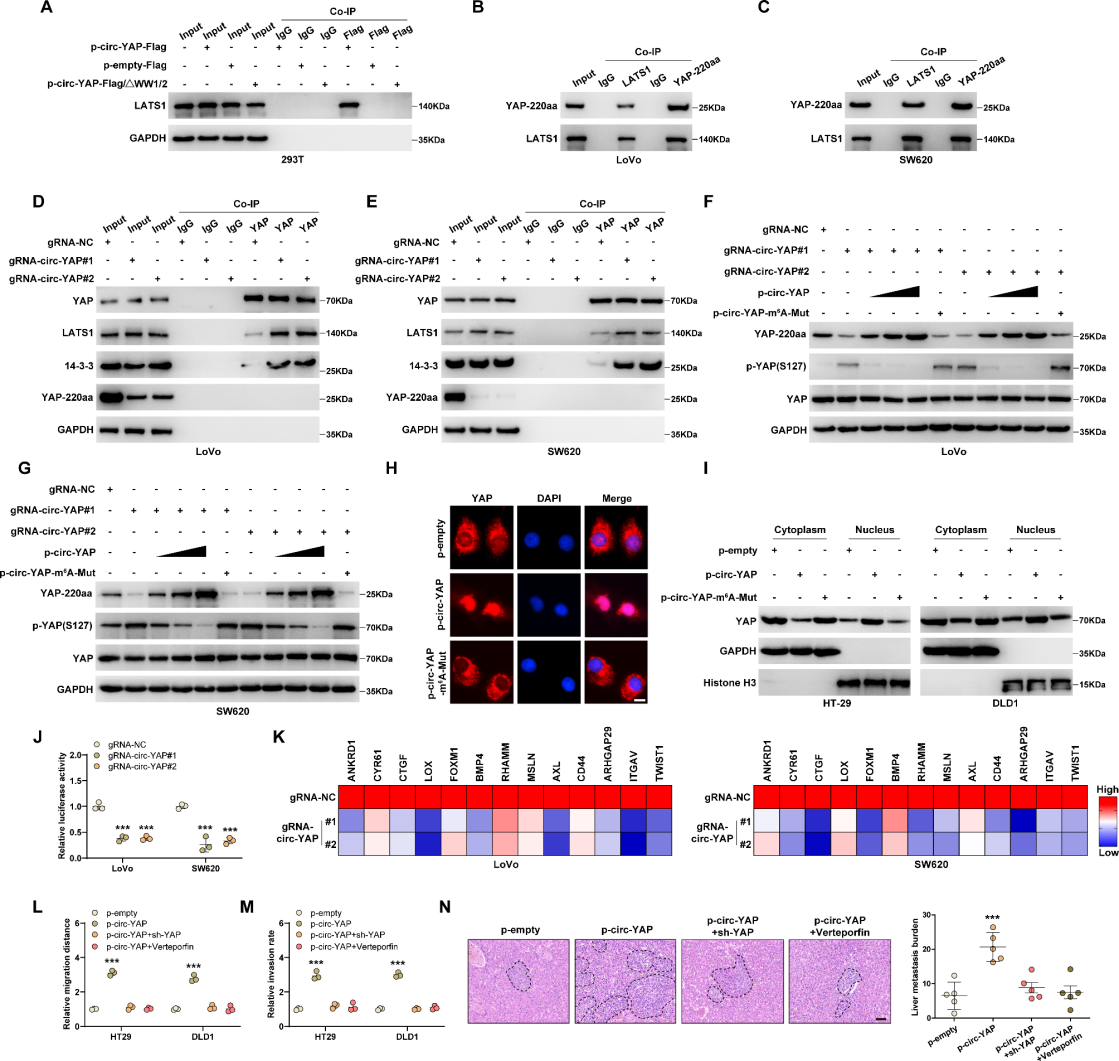
YAP-220aa increases YAP activity. **A**-**C**. Co-IP assay testing the binding of YAP-220aa to LATS1. **D**, **E**. Co-IP assay testing the interaction between YAP and LATS1/14-3-3 after circ-YAP knockdown. **F**, **G**. Western blot analysis of the indicated protein levels in circ-YAP-silenced LoVo and SW620 cells transfected with circ-YAP or circ-YAP-m^6^A-mutation. **H**, **I**. IF staining and western blot testing the location of YAP in circ-YAP-overexpressing cells. Scale bar, 25 μm. **J**. Luciferase reporter assay testing the effect of circ-YAP knockdown on YAP transcription activity in CRC cells. **K**. qRT-PCR analysis of the pro-metastasis gene expression downstream of YAP after circ-YAP knockdown. **L**, **M**. Cell migration and invasion in HT29 and DLD1 cells after YAP knockdown or verteporfin treatment. **N**. The representative images and burden of spontaneous CRC liver metastases (n = 5 per group). Scale bar, 100 μm. ***P < 0.001. Data (J, L, M) are the mean ± SD of three independent experiments carried out in triplicate. The uncropped western blot data are provided as a Original Blot Image file

Through analyzing the YAP-220aa protein sequence (Figure S5A), we found that YAP-220aa contains the WW1/2 domain of YAP, which is required for interacting with LATS1, followed by YAP phosphorylation and subsequent cytoplasmic retention by 14-3-3 [23]. Thus, we inferred that YAP-220aa might competitively bind to LATS1, blocking YAP phosphorylation. As anticipated, LATS1 was abundantly immunoprecipitated by Flag-tag antibody in HEK293T cells transfecting with p-circ-YAP-Flag vector, however, when WW1/2 domain was deleted, the above phenomenon disappeared (Fig. 5A). Further, the endogenous binding between YAP-220aa and LATS1 was found in LoVo and SW620 cells (Fig. 5B, C). Knockdown of circ-YAP markedly increased the interaction between YAP and LATS1, 14-3-3 (Fig. 5D, E). Moreover, YAP-220aa was decreased, while YAP phosphorylation was increased, in circ-YAP-silenced CRC cells, however, these effects were abrogated by wild-type circ-YAP overexpression, but not by circ-YAP overexpression with m^6^A mutation (Fig. 5F, G). Consistently, enforced expression of circ-YAP resulted in more YAP entering into the nucleus from the cytoplasm, and this phenomenon disappeared after m^6^A mutation (Fig. 5H, I). YAP luciferase reporter activity and its downstream pro-metastasis gene levels were significantly reduced in circ-YAP-silenced CRC cells (Fig. 5J, K), whereas overexpression of wild-type circ-YAP, but not overexpression of m^6^A-mutated one, exerted the opposite trend (Figure S5B, C). Phenotypically, the increased cell migration, invasion and liver metastasis nodules induced by circ-YAP were significantly abolished by YAP knockdown or treatment with verteporfin, a pharmacological inhibitor of YAP (Fig. 5L-N). Overall, these data suggest that circ-YAP-encoded YAP-220aa promotes CRC aggressiveness and liver metastasis through activating YAP via interacting with LATS1.

### YAP/TEAD complex transactivates circ-YAP

**Fig. 6:**
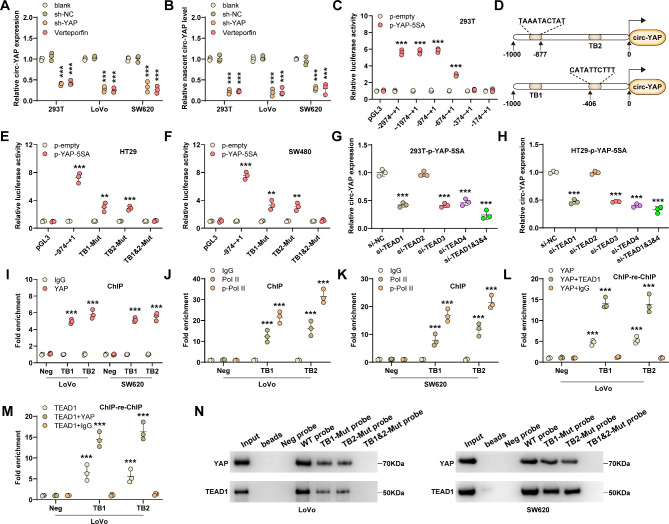
Circ-YAP is transcriptionally activated by YAP. **A**, **B**. qRT-PCR analysis of total and nascent circ-YAP levels in YAP-silenced or verteporfin-treated cells. **C**. Luciferase reporter assay testing the effect of YAP activation on circ-YAP promoter activity. **D**. Two putative YAP/TEAD binding motifs on circ-YAP promoter. **E**, **F**. The promoter activity of circ-YAP was tested in HT29 and SW480 cells co-transfected with **p**-YAP-5SA and wild-type or mutant luciferase reporter vectors. **G**, **H**. qRT-PCR analysis of circ-YAP expression in YAP-activated cells transfected with the indicated siRNAs. **I**-**K**. ChIP assay using the indicated antibodies, followed by qPCR analysis of the enrichment of YAP, PoI II and **p**-PoI II on circ-YAP promoter. **L**, **M**. ChIP-re-ChIP assay using anti-YAP/TEAD1 antibody, followed by qPCR analysis of the enrichment of YAP and TEAD1 on circ-YAP promoter. **N**. DNA pull-down assay using the wild-type or mutant biotinylated circ-YAP promoter probe, followed by western blot analysis of YAP and TEAD1 protein levels in CRC cells. *P < 0.05, **P < 0.01, ***P < 0.001. Data are the mean ± SD of three independent experiments carried out in triplicate. The uncropped western blot data are provided as a Original Blot Image file

YAP is a transcriptional co-activator that modulates gene transcription via binding to some transcription factors such as TEAD1-4 [24]. Intriguingly, YAP knockdown or verteporfin treatment dramatically reduced circ-YAP expression in both HEK293T and CRC cells (Fig. 6A). Overexpression of constitutively active YAP (YAP-5SA), but not YAP-5SA/△C (a YAP mutant lacking transactivation domain) or YAP-5SA-S94A (a YAP mutant without TEAD-binding capacity), notably increased circ-YAP levels (Figure S6A, B). Of note, the nascent circ-YAP was also affected by YAP and verteporfin (Fig. 6B, Figure S6C), indicating that YAP regulates circ-YAP expression at the transcriptional level. Emerging evidence shows that some circRNAs have their own promoters independent of their linear parents. Through analyzing ChIP-Seq data, we found that TEAD was abundantly occupied on intron 1 of YAP pre-mRNA, a potential circYAP promoter region (Figure S6D). The promoter of circYAP was fragmented and inserted into pGL3-basic vector, the luciferase results showed that YAP increased circ-YAP transcription activity at -974 ~ -374 upstream of the circ-YAP start site (Fig. 6C). Through sequence analysis using JASPAR tool, two TEAD binding motifs were found at the above region (TB1, -886 ~ -877, TAAATACTAT; TB2, -406 ~ -397, CATATTCTTT) (Fig. 6D). As shown in Fig. 6E, F, mutation of TB1 or TB2 significantly decreased the promoter activity of circ-YAP, but when mutated simultaneously, the enhanced promoter activity caused by YAP-5SA was completely counteracted. In addition, YAP-5SA increased circ-YAP expression in TEAD2-silenced cells, but not in TEAD1/3/4-silenced cells (Figure S6E, F, Fig. 6G, H), indicating that TEAD1/3/4 is responsible for YAP-mediated regulation of circ-YAP. Further, the results of ChIP assay showed that YAP bound to TB1 and TB2 (Fig. 6I), concurrently with the recruitment of Pol II and subsequent phosphorylation (Fig. 6J, K), a modification requiring for releasing Pol II from the initiation complex and starting elongation. Moreover, the ChIP-re-ChIP data showed that the YAP/TEAD1 complex was enriched at TB1 and TB2 on circ-YAP promoter (Fig. 6L, M, Figure S6G-I), which was also verified by DNA pull-down assay using biotin-labeled probes (Fig. 6N). These above results indicate that circ-YAP was transcriptionally activated by YAP, thus forming a positive feedback loop promoting CRC liver metastasis.

### Clinical verification of the circ-YAP/YAP-220aa/YAP regulatory axis

**Fig. 7:**
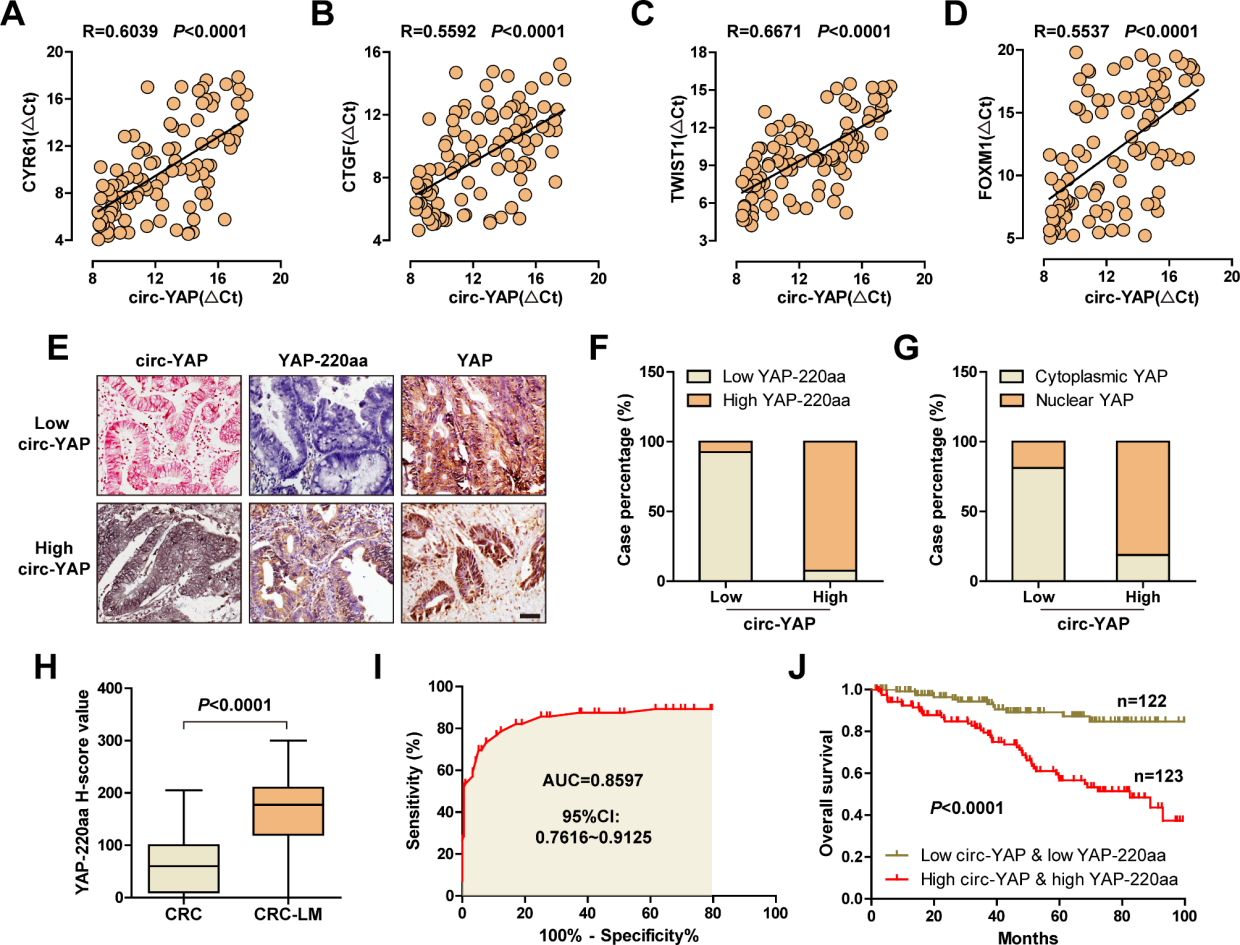
Verification of the circ-YAP/YAP-220aa/YAP regulatory axis in clinical samples. **A**-**D**. The correlations between circ-YAP expression and CYR61, CTGF, TWIST1 or FOXM1 in 100 CRC tissues. **E**-**G**. The representative ISH and IHC images of circ-YAP, YAP-220aa and YAP in CRC tissues (**E**), followed by statistical analysis (**F**, **G**). Scale bar, 100 μm. **H**. The protein expression of YAP-220aa analyzed by IHC staining in CRC tissues with liver metastasis, followed by ROC curve analysis. J. The survival curve of CRC patients with low and high circ-YAP&YAP-220aa expression

Lastly, we tested the expression of YAP target genes in CRC tissues. The qRT-PCR results showed that circ-YAP expression was positively correlated with the pro-metastasis genes including CYR61, CTGF, TWIST1 and FOXM1 (Fig. 7A-D). Moreover, the immunostaining results showed that 92.5% of CRC cases showed the same expression trend of circ-YAP and YAP-220aa, only 7.5% showed inconsistency (Fig. 7E, F). And high circ-YAP was strongly positively correlated with nuclear accumulation of YAP (Fig. 7E, G). Further, the western blot results showed that YAP-220aa and nuclear YAP were remarkably increased, while YAP phosphorylation was decreased, in CRC tissues with high circ-YAP expression (Figure S7). High YAP-220aa expression was observed in CRC with liver metastasis compared to those without liver metastasis (Fig. 7H), with an AUC value of 0.8597 (95%CI: 0.7616 ~ 0.9125) (Fig. 7I), implying that YAP-220aa may be used as an indicator to predict CRC liver metastasis. Importantly, patients with high circ-YAP&YAP-220aa displayed shorter survival time than those with low circ-YAP&YAP-220aa (Fig. 7J), indicating that combining the two parameters exerts better prognostic value than circ-YAP alone (Fig. 1J).

## Discussion

**Fig. 8:**
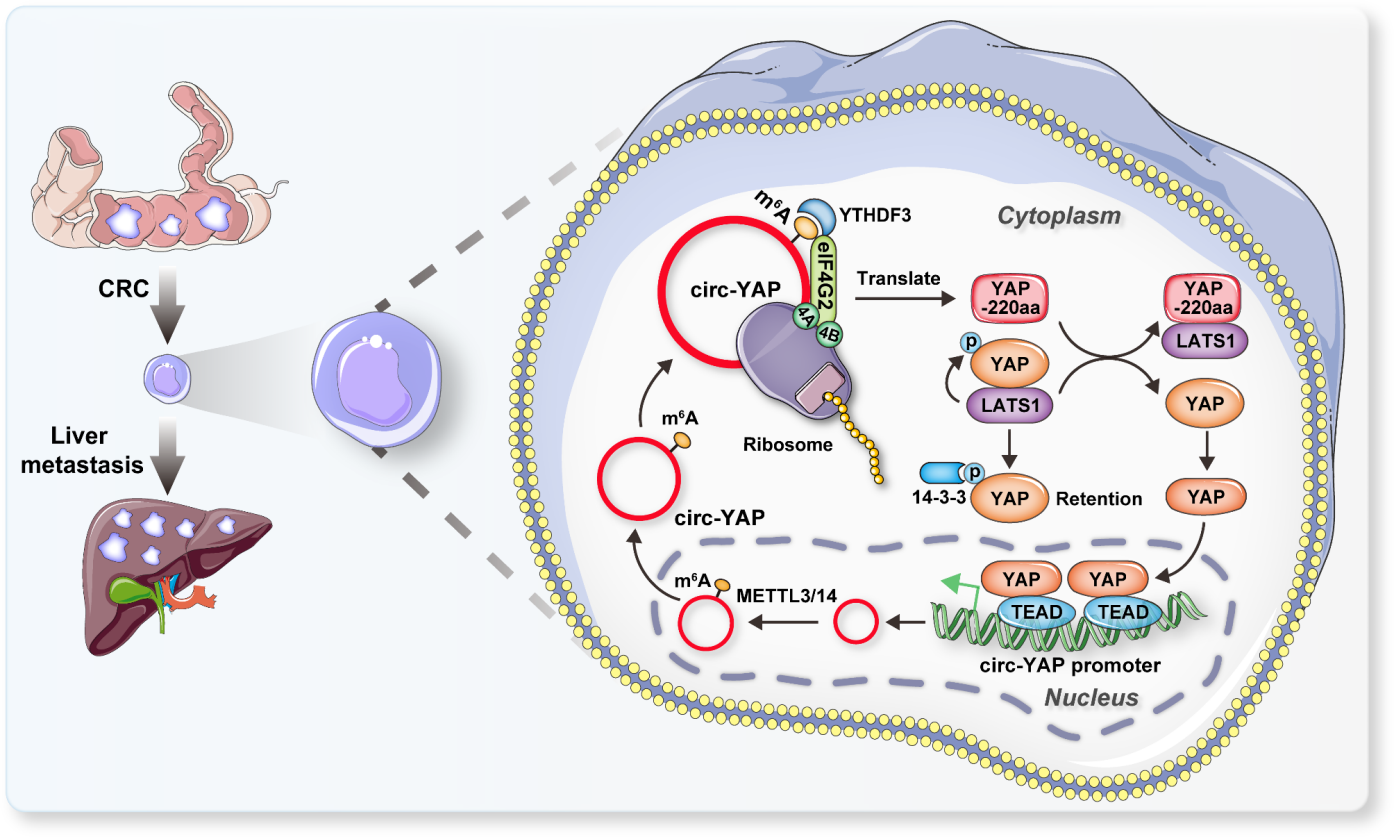
The cartoon showing the promoting effect of circ-YAP on CRC liver metastasis through translating into a novel YAP isoform, YAP-220aa, in an m^6^A-dependent manner; subsequently, YAP-220aa directly binds to LATS1 and blocks the interaction between LATS1 and YAP, resulting in YAP nuclear translocation and transcription activation of pro-metastasis genes and circ-YAP, thus forming a positive regulatory loop

CircRNA is emerging as important regulator in cancer biology, although a small number of circRNAs have been functionally characterized, many members in this class remain unexplored. Recent evidence suggests that circRNA is translatable [25], however, the roles of the circRNA-encoded proteins in human cancer remain poorly understood, let alone in CRC liver metastasis. In the present study, we for the first time identified a novel endogenous protein YAP-220aa, encoded by circ-YAP, that promoted CRC liver metastasis via blocking LATS1-mediated YAP phosphorylation and enhancing YAP activity. Stepwise investigations revealed that m^6^A reader YTHDF3 recognized m^6^A-modified circ-YAP, and recruited eIF4G2 translation initiation complex to drive circ-YAP translation. Moreover, YAP/TEAD complex was able to bind to circ-YAP promoter and activate circ-YAP transcription, thus a positive feedback circuit was formed between circ-YAP and YAP (Fig. 8). Pre-clinically, we found that circ-YAP and its translation product YAP-220aa could commendably predict CRC liver metastasis and survival time, implying a promising clinical application prospect. In aggregate, our findings provide new evidence for the important biological functions of circRNA-translated peptides, as well as new prognostic biomarker and therapeutic target for patients with CRC liver metastasis.

CircRNA regulates a wide range of biological processes in a context-dependent manner [26]. The subcellular localization is proven to be essential for circRNA function, concretely, the cytoplasmic circRNA is capable of sponging miRNAs, binding to diverse proteins and even translating into functional peptides; while the nuclear circRNA can directly interact with DNA or some transcription factors to modulate gene transcription [27]. Of note, the spatiotemporal localization of circRNA is not static, but variable, which plays the fundamental role in disease occurrence and development [28]. For instance, under stress stimuli, circ-C9ORF72 was exported from the nucleus into the cytoplasm, the nuclear export of circ-C9ORF72 accounted for neurological or neuromuscular diseases via yielding dipeptide repeat proteins [29]. In this study, we found that circ-YAP was mainly located in the cytoplasm of both normal and CRC cell lines and tissues, suggesting that the spatiotemporal localization of circ-YAP is not involved in CRC tumorigenesis. Further, circ-YAP was identified to be translated into a novel protein, YAP-220aa, and this process required m^6^A modification. As is well established, m^6^A is highly enriched in eukaryotic transcriptomes, some certain methylases, demethylases, and methylation recognition enzymes have been characterized [30]. Once theses enzymes are abnormal, a series of diseases will be driven, including tumors, neurological diseases, embryonic development retardation and so forth [31, 32]. m^6^A preferably occurs in the consensus motif “RRm^6^ACH” (R = G or A; H = A, C or U), affecting RNA stability, precursor splicing, polyadenylation, transport and translation initiation [33]. Herein, we found a highly conversed m^6^A motif “GGACA” proximal to the translation initiation site of circ-YAP, its mutation resulted in almost undetectable protein produced by circ-YAP, indicating that a single m^6^A is sufficient to drive circRNA translation, which is consistent with the previous evidence [22]. Moreover, the wild-type circ-YAP, but not the m^6^A-mutant one, promoted CRC liver metastasis, implying that circ-YAP functions in CRC depending on its translation potential. Interestingly, the expression trend of circ-YAP and YAP-220aa was not consistent in 7.5% CRC cases, this may be caused by the dysregulation of some m^6^A enzymes, or by the abnormal modification of the translation initiation complexs, etc. Additionally, although cytoplasmic localization is a prerequisite for RNA translation, 20% of circ-YAP was found in the nucleus, suggesting that circ-YAP has other roles besides coding proteins, and further investigations are needed.

The Hippo-YAP pathway is initially considered as an evolutionarily and functionally conserved modulator of organ size and growth with key roles in cell proliferation and differentiation [23]. Recently, a large body of evidence has shown that YAP is crucial for various steps of tumor metastasis, YAP binds to TEAD transcription factors to driver the formation of invasive pseudopodia, degrade extracellular matrix, induce epithelial-mesenchymal transition and maintain distant metastatic foci by regulating the target genes related to tumor metastasis and invasion [34, 35]. The Hippo core complex negatively controls the nuclear translocation of YAP, resulting in inactivation of YAP signaling, such as LATS1-mediated YAP S127 phosphorylation and subsequent cytoplasmic retention mediated by 14-3-3 protein [36]. Aberrant YAP activation has frequently been observed in metastatic cancer [37, 38], however, the underlying mechanisms are elusive and largely undetermined. In the present study, we identified a novel YAP activator, YAP-220aa, a YAP protein isoform produced by circ-YAP translation. Although YAP-220aa has a unique amino acid sequence spanning the junction site of circ-YAP, “RPVLMQALQEP”, it also contains the intact WW1/2 domain of YAP, a key domain for the interaction with LATS1, thus we inferred that YAP-220aa functioned through regulating YAP activity via competitively binding to LATS1. As expected, the endogenous binding of YAP-220aa and LATS1 was observed in CRC cells, and YAP-220aa reduced YAP phosphorylation, in conjunction with the increase of YAP nuclear translocation. Moreover, knockdown of YAP or treatment with verteporfin significantly blocked the enhanced CRC liver metastasis caused by YAP-220aa, indicating that YAP activity is crucial for the function of YAP-220aa. The expression and role of YAP-220aa in other diseases are worthy of further exploration, and whether its unique amino acid sequence forms a specific domain with function needs structural biology evidence to support. Strikingly, two YAP/TEAD binding motifs were found on the intron 1 of YAP pre-mRNA upstream of circ-YAP, mutation of them entirely abolished the increase of circ-YAP transcription caused by the YAP/TEAD complex. These suggest that circ-YAP is a novel target of YAP, the previously unrecognized positive feedback circuit amplifies the pro-CRC liver metastasis effect of circ-YAP, which also partly explains the sustained activation of YAP. Given that YAP has long been considered an “undruggable” transcription co-factor, the factors post-translationally regulating YAP may be its “Achilles’ heel“ [39]. Here, our data characterize an endogenous truncated YAP protein isoform that controls the activity of Hippo-YAP signaling, thus providing the potential therapeutic intervention for YAP-based cancer therapy.

There are several limitations in this work, with the major drawback being that the in vitro and in vivo studies were performed using human 2D cell line lines and do not truly reflect clinical tumor heterogeneity, cloning, growth and progression, the use of state-of-the-art 3D organoid systems will be helpful. In addition, our study included only retrospectively collected samples and information, the retrospective nature of the collection is associated with potential bias from variable treatments.

## Conclusions

Taken together, our study uncovers a hitherto unrecognized coupling between circ-RNA-encoded protein and Hippo/YAP signaling, with implications for the treatment of CRC liver metastasis.

### Electronic supplementary material

Below is the link to the electronic supplementary material.

Supplementary Material 1

Supplementary Material 2

Supplementary Material 3

## Data Availability

The data used to support our findings are available from the corresponding author upon reasonable request.

## Abbreviations

AUC: Area under curve
ChIP: Chromatin immunoprecipitation
CircRNA: Circular RNA
Co-IP: Co-immunoprecipitation
CRC: Colorectal cancer
FISH: Fluorescence in situ hybridization
IHC: Immunohistochemisty
m^6^A: N6-methyladenosine
ORF: Open reading frame
PDX: Patient-derived xenografts
qRT-PCR: Quantitative real-time polymerase chain reaction

## References

[1] Sung H, Ferlay J, Siegel RL (2021). Global Cancer Statistics 2020: GLOBOCAN estimates of incidence and Mortality Worldwide for 36 cancers in 185 countries. CA Cancer J Clin.

[2] Osei-Bordom DC, Kamarajah S, Christou N. Colorectal Cancer, Liver Metastases and Biotherapies. Biomedicines. 2021, 9. 10.3390/biomedicines9080894 PMC8389538 34440099

[3] Tsilimigras DI, Brodt P, Clavien PA (2021). Liver metastases. Nat Rev Dis Primers.

[4] Patop IL, Wust S, Kadener S (2019). Past, present, and future of circRNAs. EMBO J.

[5] Kristensen LS, Andersen MS, Stagsted L, Ebbesen KK, Hansen TB, Kjems J (2019). The biogenesis, biology and characterization of circular RNAs. Nat Rev Genet.

[6] Chen X, Zhou M, Yant L, Huang C (2022). Circular RNA in disease: Basic properties and biomedical relevance. Wiley Interdiscip Rev RNA.

[7] Lei M, Zheng G, Ning Q, Zheng J, Dong D (2020). Translation and functional roles of circular RNAs in human cancer. Mol Cancer.

[8] Kristensen LS, Jakobsen T, Hager H, Kjems J (2022). The emerging roles of circRNAs in cancer and oncology. Nat Rev Clin Oncol.

[9] Liu CX, Chen LL (2022). Circular RNAs: characterization, cellular roles, and applications. Cell.

[10] Memczak S, Jens M, Elefsinioti A (2013). Circular RNAs are a large class of animal RNAs with regulatory potency. Nature.

[11] Hansen TB, Jensen TI, Clausen BH (2013). Natural RNA circles function as efficient microRNA sponges. Nature.

[12] Huang A, Zheng H, Wu Z, Chen M, Huang Y (2020). Circular RNA-protein interactions: functions, mechanisms, and identification. Theranostics.

[13] Wang Y, Wu C, Du Y (2022). Expanding uncapped translation and emerging function of circular RNA in carcinomas and noncarcinomas. Mol Cancer.

[14] He L, Man C, Xiang S, Yao L, Wang X, Fan Y (2021). Circular RNAs’ cap-independent translation protein and its roles in carcinomas. Mol Cancer.

[15] Du WW, Xu J, Yang W (2021). A neuroligin isoform translated by circNlgn contributes to Cardiac Remodeling. Circ Res.

[16] Gao X, Xia X, Li F (2021). Circular RNA-encoded oncogenic E-cadherin variant promotes glioblastoma tumorigenicity through activation of EGFR-STAT3 signalling. Nat Cell Biol.

[17] Li Y, Chen B, Zhao J (2021). HNRNPL Circularizes ARHGAP35 to produce an oncogenic protein. Adv Sci (Weinh).

[18] Wen SY, Qadir J, Yang BB (2022). Circular RNA translation: novel protein isoforms and clinical significance. Trends Mol Med.

[19] Jiang X, Liu B, Nie Z (2021). The role of m6A modification in the biological functions and diseases. Signal Transduct Target Ther.

[20] Zeng K, Chen X, Hu X (2018). LACTB, a novel epigenetic silenced tumor suppressor, inhibits colorectal cancer progression by attenuating MDM2-mediated p53 ubiquitination and degradation. Oncogene.

[21] Li S, Li X, Xue W (2021). Screening for functional circular RNAs using the CRISPR-Cas13 system. Nat Methods.

[22] Yang Y, Fan X, Mao M (2017). Extensive translation of circular RNAs driven by N(6)-methyladenosine. Cell Res.

[23] Moya IM, Halder G (2019). Hippo-YAP/TAZ signalling in organ regeneration and regenerative medicine. Nat Rev Mol Cell Biol.

[24] Ibar C, Irvine KD (2020). Integration of Hippo-YAP signaling with metabolism. Dev Cell.

[25] Kong S, Tao M, Shen X, Ju S (2020). Translatable circRNAs and lncRNAs: driving mechanisms and functions of their translation products. Cancer Lett.

[26] Plawgo K, Raczynska KD. Context-dependent regulation of Gene expression by Non-Canonical Small RNAs. Noncoding RNA. 2022, 8. 10.3390/ncrna8030029 PMC9149963 35645336

[27] Misir S, Wu N, Yang BB (2022). Specific expression and functions of circular RNAs. Cell Death Differ.

[28] Yang Q, Li F, He AT, Yang BB (2021). Circular RNAs: expression, localization, and therapeutic potentials. Mol Ther.

[29] Wang S, Latallo MJ, Zhang Z (2021). Nuclear export and translation of circular repeat-containing intronic RNA in C9ORF72-ALS/FTD. Nat Commun.

[30] Deng LJ, Deng WQ, Fan SR (2022). m6A modification: recent advances, anticancer targeted drug discovery and beyond. Mol Cancer.

[31] Zhang F, Liu H, Duan M (2022). Crosstalk among m(6)a RNA methylation, hypoxia and metabolic reprogramming in TME: from immunosuppressive microenvironment to clinical application. J Hematol Oncol.

[32] Frye M, Harada BT, Behm M, He C (2018). RNA modifications modulate gene expression during development. Science.

[33] Niu X, Yang Y, Ren Y, Zhou S, Mao Q, Wang Y. Crosstalk between m(6)a regulators and mRNA during cancer progression. Oncogene. 2022. 10.1038/s41388-022-02441-4 36008465

[34] Li HL, Li QY, Jin MJ (2021). A review: hippo signaling pathway promotes tumor invasion and metastasis by regulating target gene expression. J Cancer Res Clin Oncol.

[35] Zanconato F, Cordenonsi M, Piccolo S (2019). YAP and TAZ: a signalling hub of the tumour microenvironment. Nat Rev Cancer.

[36] Zhao B, Li L, Tumaneng K, Wang CY, Guan KL (2010). A coordinated phosphorylation by lats and CK1 regulates YAP stability through SCF(beta-TRCP). Genes Dev.

[37] Warren J, Xiao Y, Lamar JM. YAP/TAZ activation as a target for treating Metastatic Cancer. Cancers (Basel). 2018. 10. 10.3390/cancers10040115 PMC5923370 29642615

[38] Tocci P, Blandino G, Bagnato A (2021). YAP and endothelin-1 signaling: an emerging alliance in cancer. J Exp Clin Cancer Res.

[39] Yan F, Qian M, He Q, Zhu H, Yang B (2020). The posttranslational modifications of Hippo-YAP pathway in cancer. Biochim Biophys Acta Gen Subj.

