# Supplementary material for: A positive feedback circuit driven by m6A-modified circular RNA facilitates colorectal cancer liver metastasis
Source: Mol Cancer. 2023 Dec 13;22:202. doi: 10.1186/s12943-023-01848-1 (PMC10717141; doi:10.1186/s12943-023-01848-1)
Supplement: Supplementary file 1 — Supplementary Material 1 [file 12943_2023_1848_MOESM1_ESM.doc]

**Table S1. The primer sequences used in this study**

| **Gene** | **Sequence (5`-3`)** |
| --- | --- |
| Circ-YAP-F | ATGGTGGGACTCAAAATCCA |
| Circ-YAP-R | GAGAAACAGCTCCCAACTGC |
| YAP mRNA-F | AATCCCACTCCCGACAGG |
| YAP mRNA-R | GACTACTCCAGTGGGGGTCA |
| ANKRD1 mRNA-F | GCCTACGTTTCTGAAGGCTG |
| ANKRD1 mRNA-R | GTGGATTCAAGCATATCACGGAA |
| CYR61 mRNA-F | AAGAAACCCGGATTTGTGAG |
| CYR61 mRNA-R | GCTGCATTTCTTGCCCTTT |
| CTGF mRNA-F | TGCTTTGAACGATCAGACAA |
| CTGF mRNA-R | CTTGTGGCAAGTGAATTTCC |
| LOX mRNA-F | GCCGACCAAGATATTCCTGGG |
| LOX mRNA-R | GCAGGTCATAGTGGCTAAACTC |
| FOXM1 mRNA-F | TGCAGCTAGGGATGTGAATCTTC |
| FOXM1 mRNA-R | GGAGCCCAGTCCATCAGAACT |
| BMP4 mRNA-F | ACGGTGGGAAACTTTTGATGTG |
| BMP4 mRNA-R | CGAGTCTGATGGAGGTGAGTC |
| RHAMM mRNA-F | ACCTTCAGTTTCTGGAGCTGG |
| RHAMM mRNA-R | GGAGATGGTGCACAACCAGA |
| MSLN mRNA-F | GGACTTGGCCACGTTCATG |
| MSLN mRNA-R | ACCTCAGCCACAGTCAACGG |
| AXL mRNA-F | CAGCGCAGCCTGCATGT |
| AXL mRNA-R | TTGGCGTTATGGGCTTCG |
| CD44 mRNA-F | ACCGACAGCACAGACAGAATC |
| CD44 mRNA-R | GTTTGCTCCACCTTCTTGACTC |
| ARHGAP29 mRNA-F | CCTTGCAAAAGAGATCCAACA |
| ARHGAP29 mRNA-R | TTGATGCTGGCAATTGTCTT |
| ITGAV mRNA-F | ATCTGTGAGGTCGAAACAGGA |
| ITGAV mRNA-R | TGGAGCATACTCAACAGTCTTTG |
| TWIST1 mRNA-F | GGAGTCCGCAGTCTTACGAG |
| TWIST1 mRNA-R | TCTGGAGGACCTGGTAGAGG |
| GAPDH mRNA-F | TGTTGCCATCAATGACCCCTT |
| GAPDH mRNA-R | CTCCACGACGTACTCAGCG |
