# Supplementary material for: A positive feedback circuit driven by m6A-modified circular RNA facilitates colorectal cancer liver metastasis
Source: Mol Cancer. 2023 Dec 13;22:202. doi: 10.1186/s12943-023-01848-1 (PMC10717141; doi:10.1186/s12943-023-01848-1)
Supplement: Supplementary file 2 — Supplementary Material 2 [file 12943_2023_1848_MOESM2_ESM.pdf]

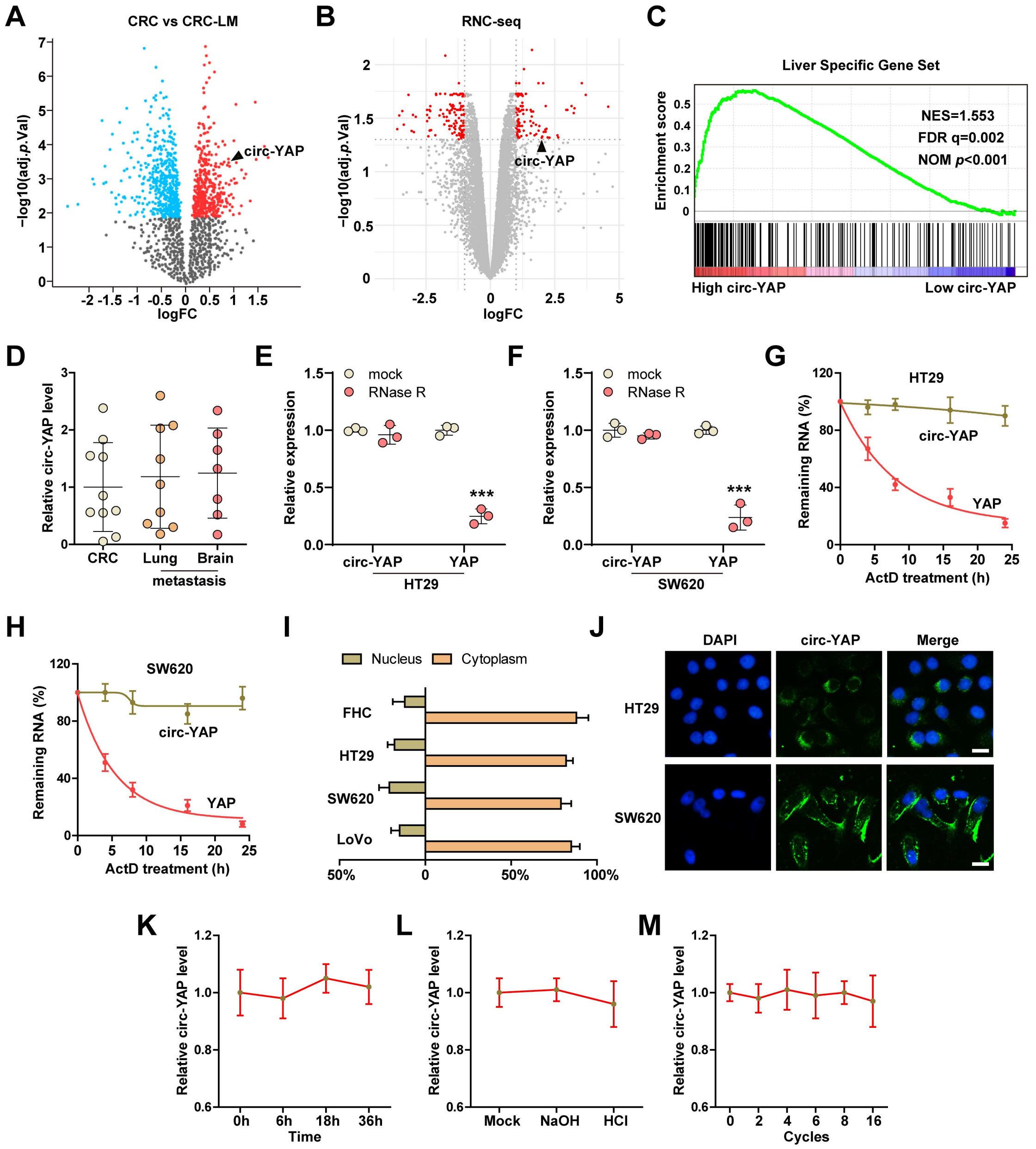

Figure S1. A, B. The circRNA microarray and ribosome nascent-chain complex-bound RNA sequencing were used to identify the key circRNAs with translational activity in CRC liver metastasis. C. GESA enrichment analysis detecting the correlation between circ-YAP and liver specific gene set. D. qRT-PCR analysis of circ-YAP in CRC tissues with lung or brain metastasis. E-H. Cells were treated with 3U/μg of RNase R or 5μg/ml Actinomycin D, followed by qRT-PCR analysis of circ-YAP and YAP mRNA levels. I, J. qRT-PCR and FISH detecting the location of circ-YAP in the indicated cells. Scale bar, 25μm. K-M. qRT-PCR analysis testing the stability of circ-YAP under the indicated conditions. ***P<0.001. Data are the mean±SD of three independent experiments carried out in triplicate.

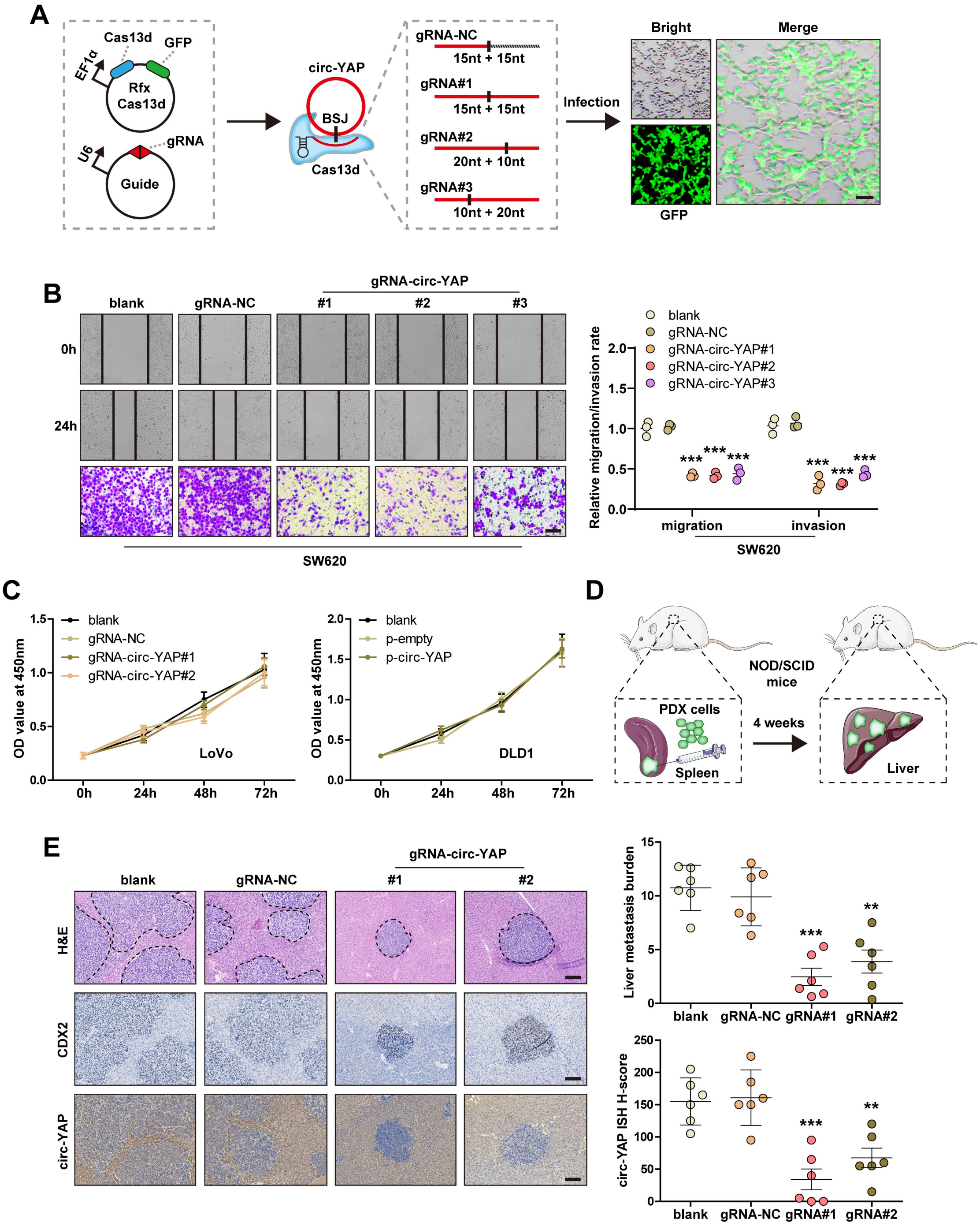

Figure S2. A. The sketch showing the technical process of CRISPR/Cas13d-mediated circ-YAP knockdown. Scale bar, 50μm. B. Cell migration and invasion in SW620 cells with circ-YAP knockdown. Scale bar, 100μm. C. CCK-8 assay testing cell viability in LoVo and DLD1 cells after circ-YAP knockdown or overexpression. D. The sketch showing the establishment of the experimental liver metastasis model. E. The representative images of CRC liver metastasis, IHC staining of CDX2, ISH staining of circ-YAP in the indicated groups. Scale bar, 100μm. $^{**}P<0.01$, $^{***}P<0.001$. Data are the mean±SD of three independent experiments carried out in triplicate.

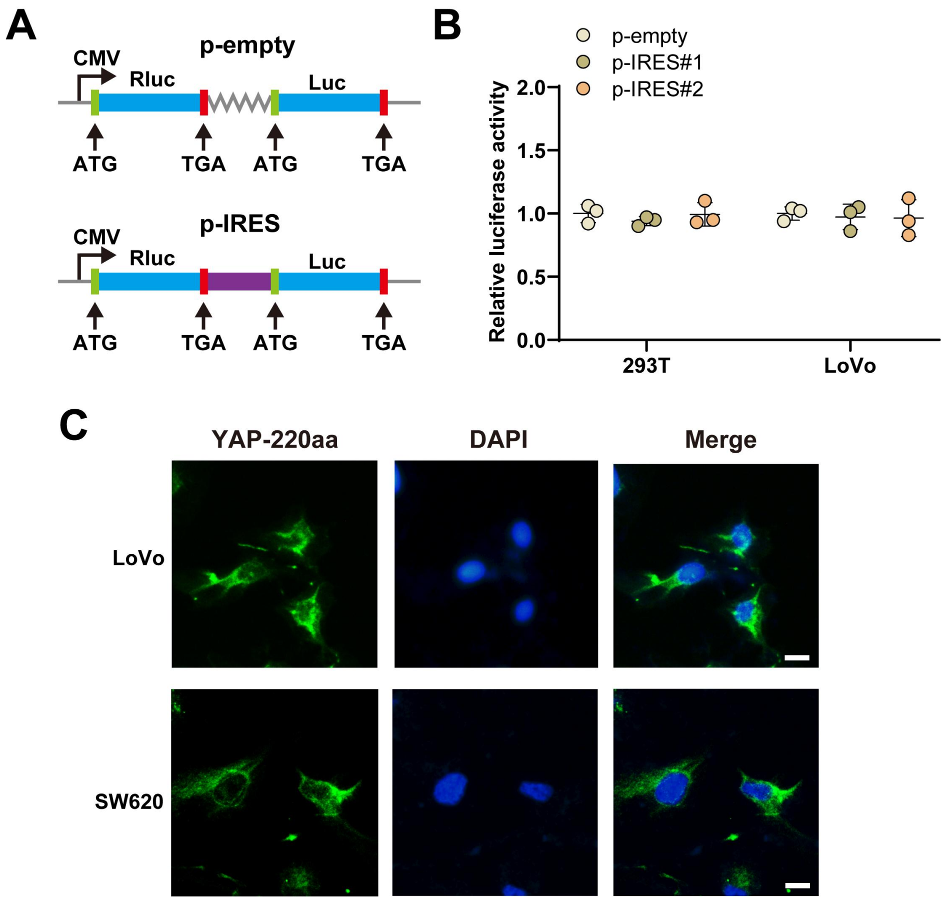

Figure S3. A, B. The sketch showing the luciferase vector construction, followed by luciferase reporter assay testing the translation activities of the putative IRES sequences in 293T and LoVo cells. C. IF staining using anti-YAP-220aa antibody in LoVo and SW620 cells, cell nucleus was stained by DAPI. Scale bar, 25μm. Data are the mean±SD of three independent experiments carried out in triplicate.

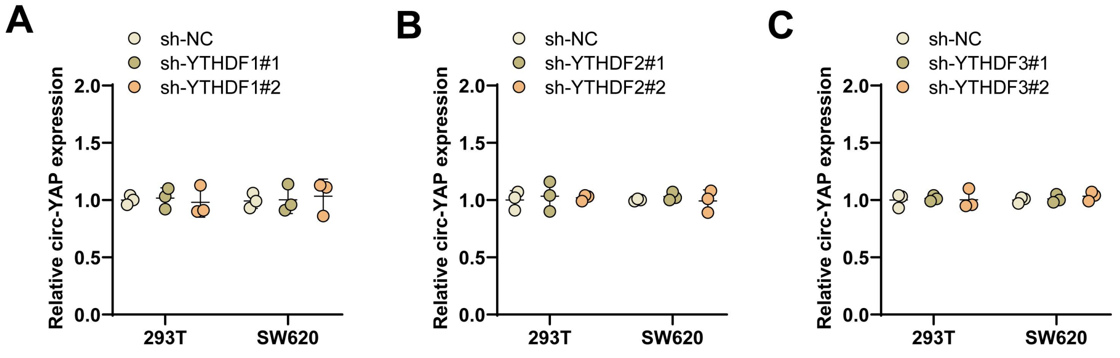

Figure S4. A-C. qRT-PCR analysis of circ-YAP expression in 293T and SW620 cells with YTHDF1/2/3 knockdown. Data are the mean±SD of three independent experiments carried out in triplicate.

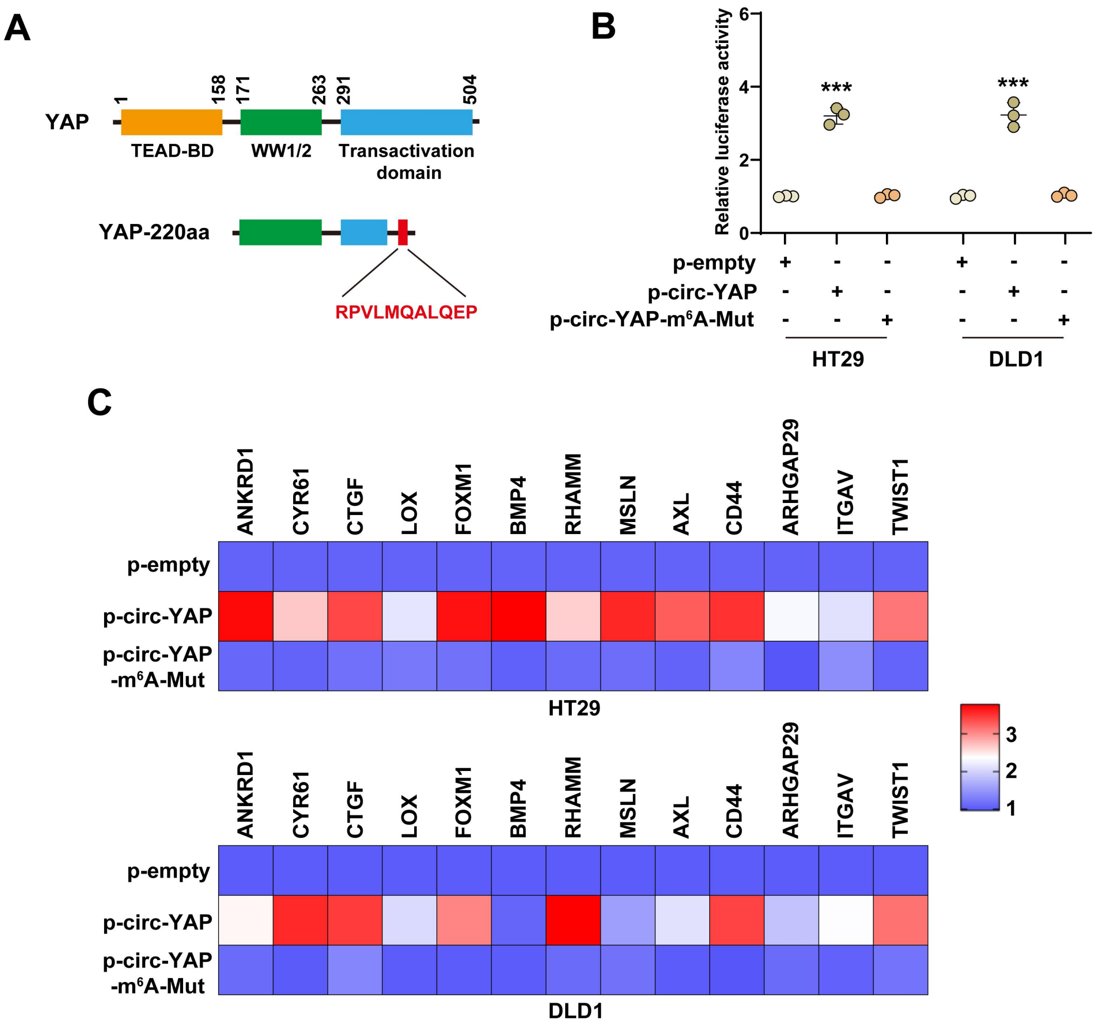

Figure S5. A. The sketch showing the domains of YAP and YAP-220aa proteins. B, C. HT29 and DLD1 cells were transfected with wild-type or m^{6}A-mutated circ-YAP expression vector, followed by luciferase reporter assay testing the transcription activity of YAP (B), and qRT-PCR analysis of pro-metastasis gene expression downstream of YAP (C). ****P*<0.001. Data are the mean±SD of three independent experiments carried out in triplicate.

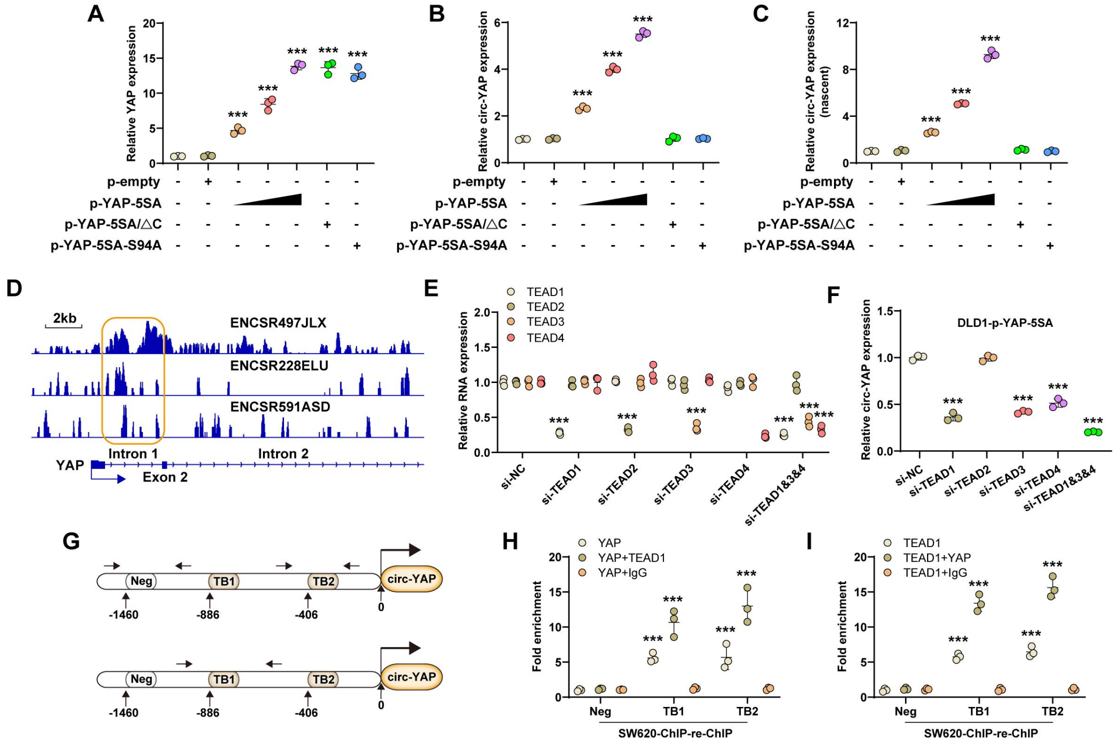

Figure S6. A-C. qRT-PCR analysis of YAP, circ-YAP and nascent circ-YAP in cells transfected with the indicated vectors. D. ChIP-seq data showing the enrichment of TEAD1 on YAP Intron 1. E. qRT-PCR analysis verifying the silencing effects of the indicated siRNAs. F. qRT-PCR analysis of circ-YAP expression in YAP-activated cells transfected with the indicated siRNAs. G- I. The schematic diagram of primer design (G), followed by ChIP-re-ChIP assay using anti-YAP/TEAD1 antibody and qPCR analysis of the enrichment of YAP and TEAD1 on circ-YAP promoter. ****P*<0.001. Data are the mean±SD of three independent experiments carried out in triplicate.

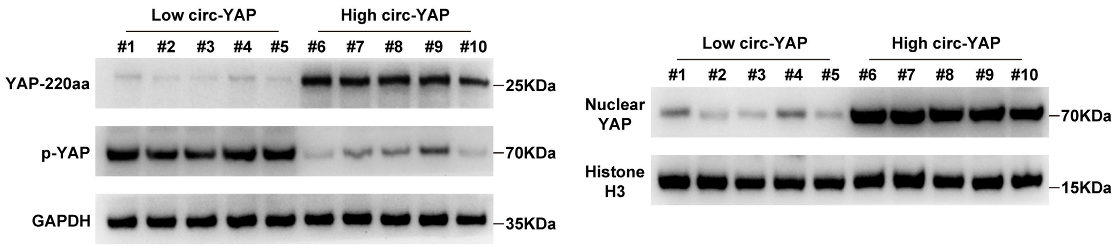

Figure S7. Western blot analysis of YAP-220aa, p-YAP and nuclear YAP protein levels in 10 CRC fresh tissues (5 cases with low circ-YAP, 5 cases with high circ-YAP). GAPDH and Histone H3 were used as loading controls for cytoplasmic and nuclear fragments, respectively. The uncropped western blot data are provided as a Original Blot Image file.
