# Supplementary figures and images for: A positive feedback circuit driven by m6A-modified circular RNA facilitates colorectal cancer liver metastasis
Source: Mol Cancer. 2023 Dec 13;22:202. doi: 10.1186/s12943-023-01848-1 (PMC10717141; doi:10.1186/s12943-023-01848-1)

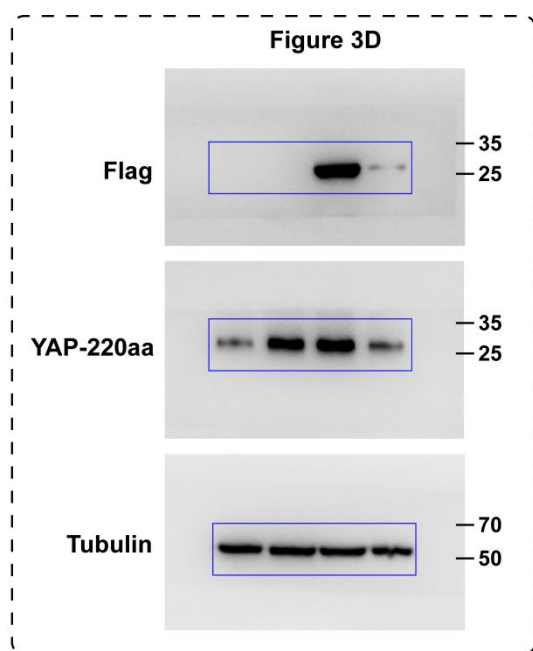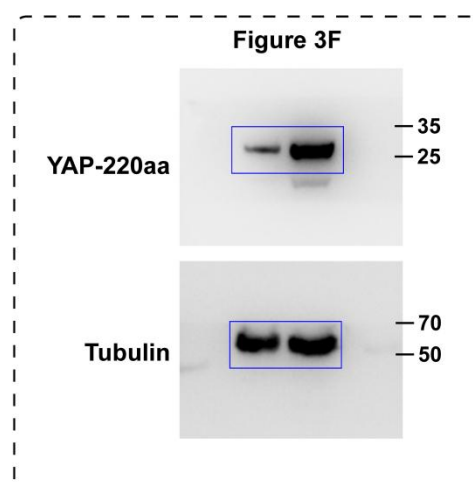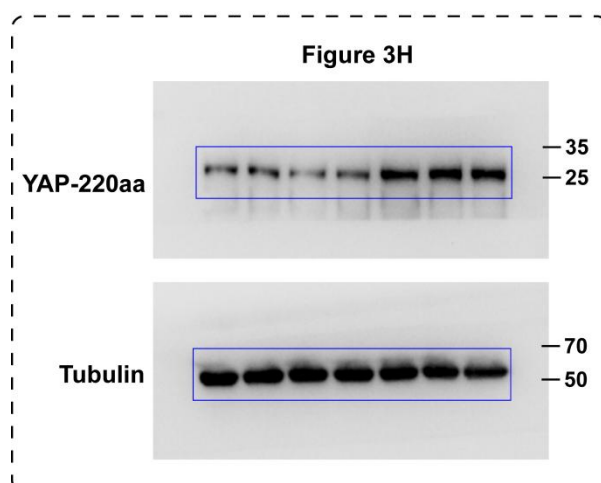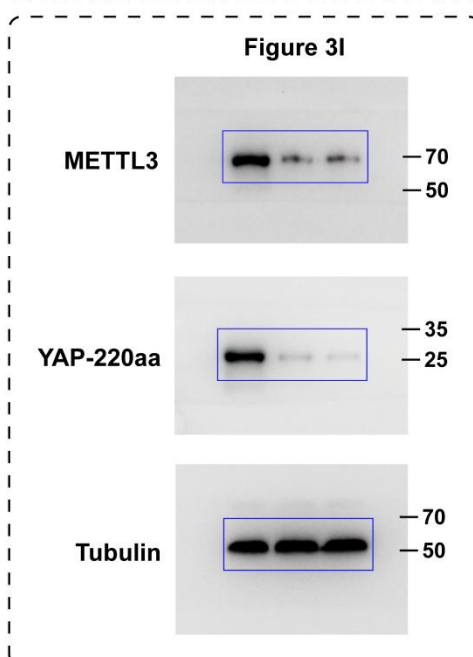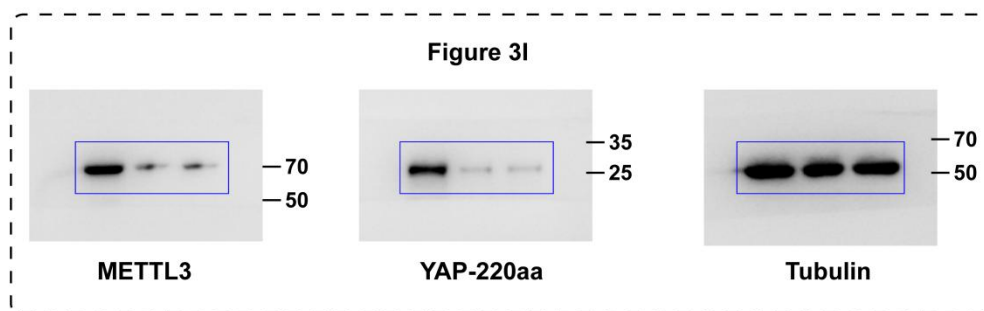

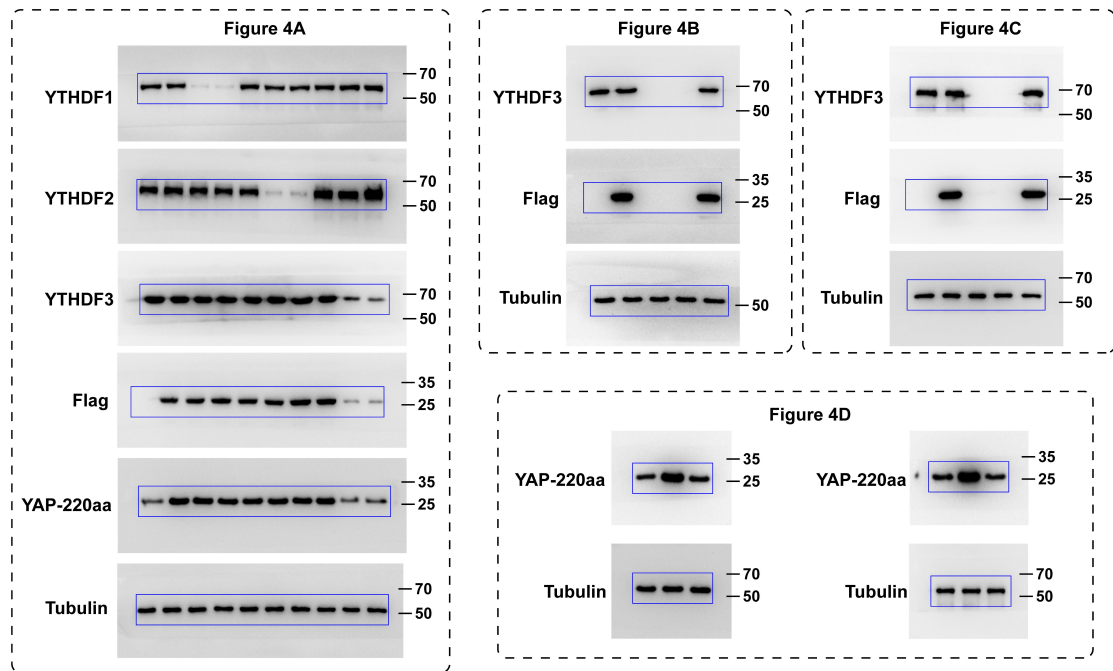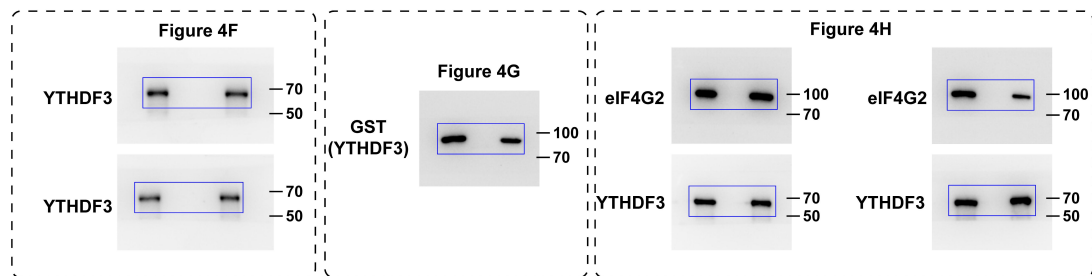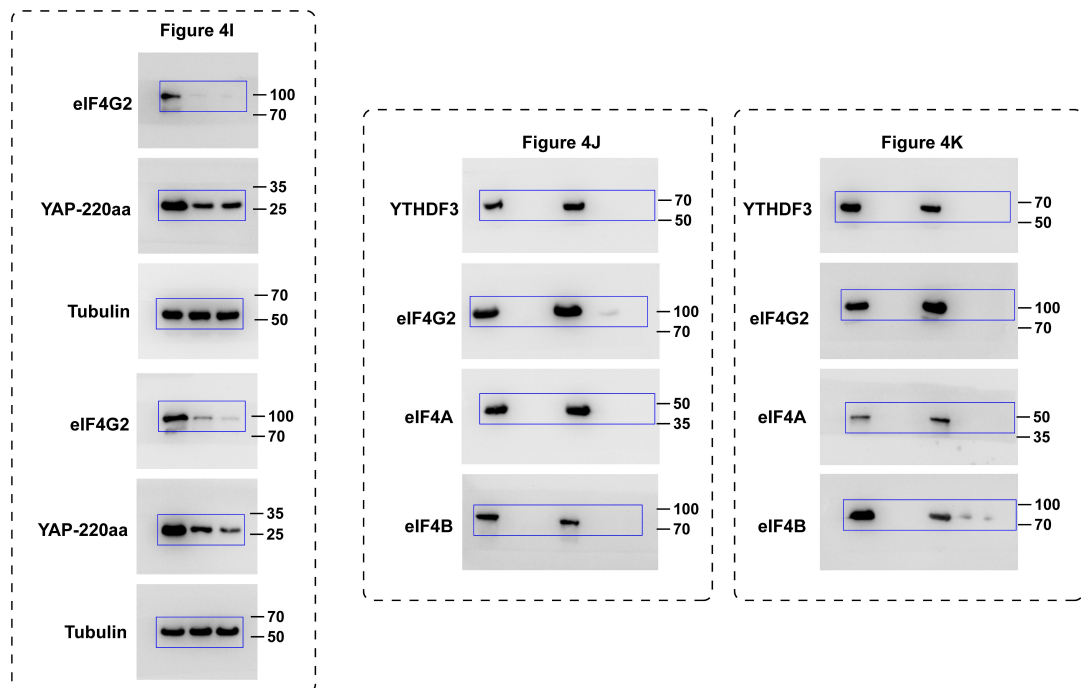

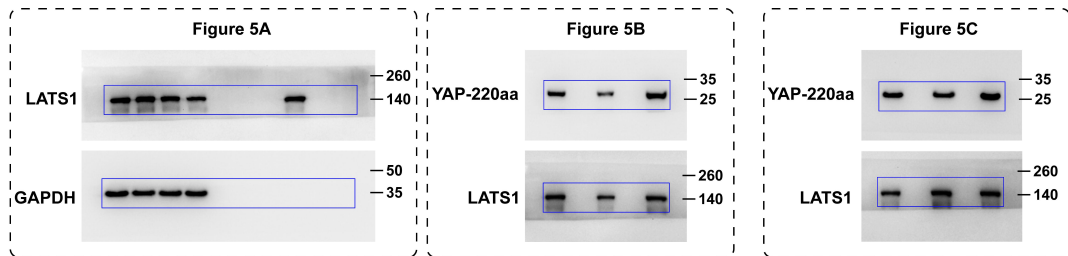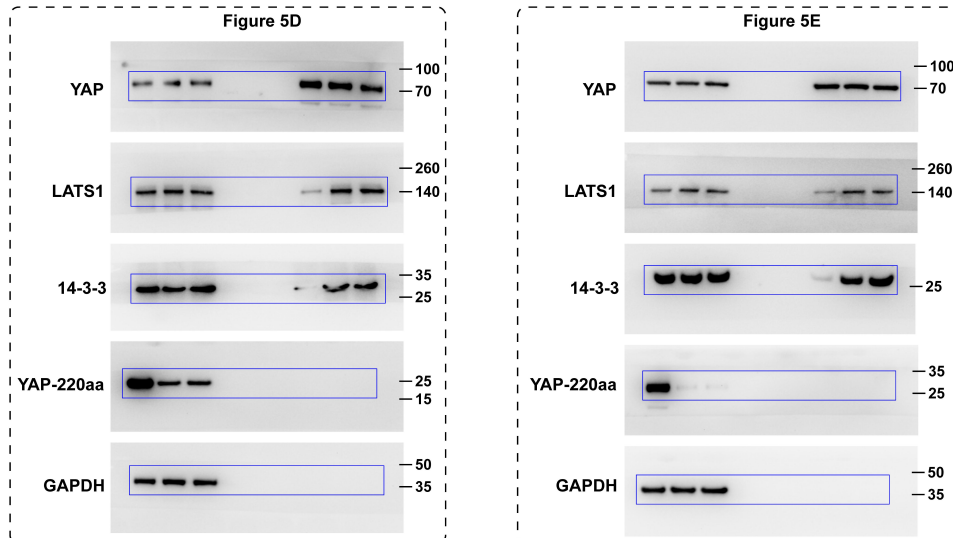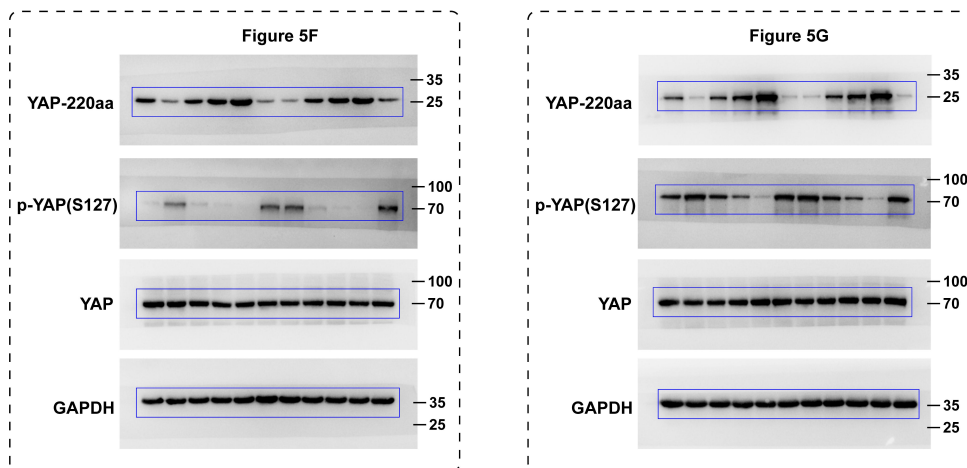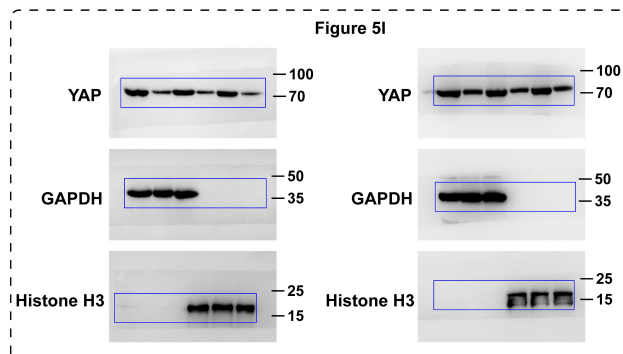

Figure 6N

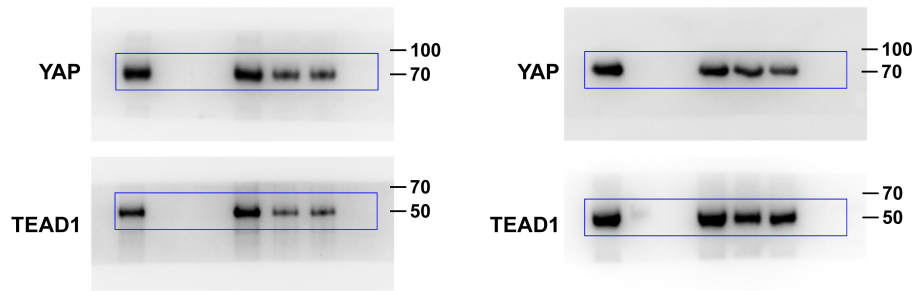

Figure 7

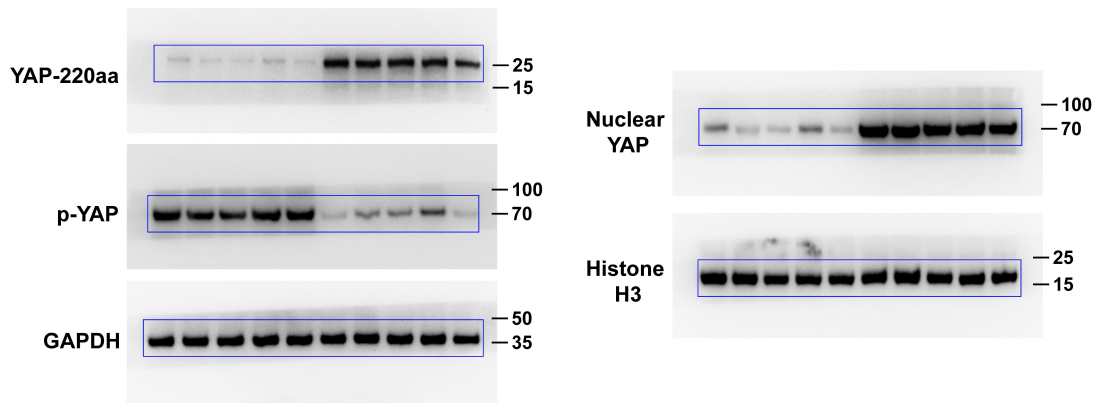

Supplement: Supplementary file 3 — Supplementary Material 3 [file 12943_2023_1848_MOESM3_ESM.pdf]
